# Clinical Review, and Hospital Reports

**Published:** 1837-04-01

**Authors:** 


### Clinical Review.

### North London Hospital.

### The Best Mode of Paring a Bottle-Nose.

It is well known to the " men about town," that Mr. Liston has operated on several noses, the protuberance and tint of which had acquired their wearers a reputation not altogether pleasant. One or two gentlemen had felt to their cost the truth of Coleman's song, that:—

> Though every one can't have a beautiful nose,

> Still, a nose shouldn't look like a snout.

Hypertrophy of the nose, it is scientifically termed lipoma, is more prevalent, perhaps, in the upper than in the lower classes. It is a gentlemanly sort of malady, and usually demonstrates that the patient has not entertained an insuperable antipathy to " port." Sheridan and Sir William Curtis had each of them a nose much larger than what usually falls to the lot of men, and of a very gorgeous purple tint. They were excellent good fellows, and bore their blushing honours on them to the last. Yet we doubt whether they would not now (could they be recalled) avail themselves of the scalpel of Mr. Liston, who has been fashioning noses, both of Grecian and of Roman cut, according to the predilections of the parties. We extract the following clinical observations on the disease from the Lancet.* They give a better account of it, and of the operation required for its removal, than we have elsewhere seen. Passing over some references to authors who have treated of it, we proceed to Mr. Liston's own observations.

" He considered that the disease affected the cellular tissue in many cases more than the skin, being a sort of hypertrophy of the subcutaneous cellular tissue; the cartilages may, perhaps, be a little thinned, but are not involved in the disease; the skin is thickened, and opened out in its texture, and the sebaceous follicles are very much enlarged. They were not, however, so large in this as he had seen them in several other cases which had been under his care. The internal structure resembled the Barbadoes leg, and the tumour which sometimes appears in the scrotum. They saw in this preparation taken from the patient Snell that the disease was seated in the cellular tissue; the size of the tumour had, however, much diminished since its removal by operation, in consequence of the escape of a large quantity of albuminous serosity which it contained. He had described the disease some years since under the title of lipoma of the nose, and he might read a short passage from his *Elements of Surgery* descriptive of it:— ' The integuments covering the apex and alæ of the nose are sometimes opened out in texture by interstitial deposit, forming a lipomatous tumour, lobulated, discoloured, and intersected by fissures. The sebaceous follicles are sometimes enlarged enormously, so as to admit the point of a quill. Turgid veins ramify superficially, and the surface is of a reddish blue or a purple colour, varying its hue from time to time according to the state of the health, and the changes in the circulation. The enlargement often attains great magnitude, producing much deformity; vision is obstructed, and the introduction of food, both solid and liquid, interfered with; the lobes tumble into the wine-glass, spoon, and cup, and are sometimes so elongated as to require being pulled aside in order to uncover the mouth; breathing is also impeded, more or less, by encroachment on the nasal orifices. The disease may be often attributed to hard living, but many not intemperate labour under it.

" It is desirable to have the tumour removed, even before it has become large; and it can readily be conceived that local applications must fail in

* Lancet, Jan. 21, 1837.

bringing the skin into a healthy condition. Incision is required. If both sides of the nose are affected, a small scalpel is carried down in the mesial line through the altered structure, and whilst an assistant places his finger in the nostril the surgeon lays hold of the integuments with a sharp hook, and carefully dissects away the diseased parts, first on one side and then on the other. The vessels are then tied, and sometimes a considerable number bleed smartly; oozing may continue, but is readily commanded by continued pressures, the nostrils being well stuffed. Afterwards such dressings may be applied as agree with the stages of the sore. After cicatrization the comfort and appearance of the patient are much enhanced, and there is no risk of reproduction.' To this description is appended a note, which states, that in making a section of the tumour accumulations of sebaceous matter are sometimes observed. An accumulation may be perceived in one of the follicles, forming an encysted tumour nearly as large as a nut, in one of these specimens, which was removed some months since."

Mr. L. has never known lipoma end in malignant change of structure. The great objections to it are the inconveniences that it occasions, and particularly the unfavourable impression which it makes on lookers-on. No body likes to be hooted at by the little boys, and to be called "nosey" in the streets.

The use of the ligature has been recommended. But it is painful—disgusting from the stench occasioned by the slough—and it does not satisfactorily remove the deformity. It is only in very vascular tumors that it should be used, in combination, or not, with excision, according to the circumstances of the case. We extract some further hints upon the mode of operating.

"The operation by incision is not attended with great difficulty, the hæmorrhage is readily restrained by pressure, the nostrils being plugged. You need not stop to tie the vessels during the dissection; afterwards it may be necessary to tie one or two. You would notice how the sides of the nose were cleared in this case; an assistant placed one finger on the nostrils, so that the cartilages of the apex and the alæ might not be encroached upon. The best plan is to cut right down in the mesial line, and then to dissect away the diseased mass, first from one side and then from the other, keeping the two sides as like as possible to each other. He had been in the habit of operating, and had removed in this way a great many tumours, without any unpleasant symptoms following. It might be supposed that after removal of the integuments cicatrization might be very slow, and that the cicatrix would be discoloured; in this they would be agreeably disappointed, the healing process going on very rapidly: the scar may remain for a short time a little red and tender, but it ultimately comes to resemble closely the surrounding skin. The countenances of these patients are generally of a rubicund cast, which is so far favourable as regards the appearance afterwards."

In only one case had Mr. Liston been called on to perform a second operation. In that case incipient disease in the alæ was left, and in the course of ten years it had acquired such dimensions as to require removal.

---

### Glasgow Royal Infirmary.

### Fracture of the Neck of the Thigh-Bone external to the Capsule.

Mr. Douglas has made some observations in the last number of our contemporary of Dublin,* the object of which is to demonstrate the occurrence of complete bony union of the neck of the thigh-bone, external to the capsular ligament. He relates two cases, but as most surgeons are not disposed to question the fact, one will be sufficient for the purpose.

*Case.* "An elderly man was admitted into the surgical wards of the Glasgow Royal Infirmary, on account of fracture of the neck of the femur,

* March, 1837.

external to the capsule. His limb was put up in Desault's splints, as is the constant practice in that hospital in all fractures of the thigh, and at the end of six weeks it was found firm. Still on rotation a crepitus was perceived at the inner part of the thigh-bone, and a suspicion being excited that all was not right, the splints were re-applied, and after a considerable time he was dismissed, because able to walk, although this crepitus had not disappeared. Shortly after this he was brought up to the wards of which I had charge, as physician's clerk, *in articulo mortis*, it was said from cholera, and died immediately after being put to bed. Learning that this was the same individual who had been above stairs with the fractured thigh, I speedily possessed myself of his femur, the state of which is as follows:—

The bone has been split into four pieces, the neck being detached from the shaft immediately beyond the inter-trochanteric lines, and depressed about half an inch below its proper level, though still retaining its natural obliquity. The greater trochanter is split from above and before downwards and backwards, and the loose portion is thrown a little inwards upon the neck. The lesser trochanter is drawn a little upwards and forwards. The fractured surfaces of the neck, shaft, and great trochanter, are firmly united by bone, except at the top of the great trochanter, where an opening exists between the two pieces, leading downwards into the medullary cavity. The trochanter minor is supported by a buttress of new bone, to which it is connected by ligament only, and on which it is still moveable. It was this motion which produced the crepitus after the other fractures were united, and led to the re-application of the splints. The preparation is preserved in my museum, and bears a striking resemblance to the specimen figured by Mr. Colles in the Dublin Hospital Reports, vol. ii. fig. 3, only that the neck is not driven across the shaft."

---

### Western Lying-in Hospital.

### On the Length of the Umbilical Cord and its Mechanical Influence upon Parturition.*

This is a paper of a statistical character, and a very good one. The author, Dr. Churchill, physician to the Western Lying-in Hospital, first notices the different, indeed contradictory, statements of preceding writers, with respect to the length of the umbilical cord. These statements we need not advert to, but content ourselves with the facts collected by the industry of Dr. Churchill himself. We will not, however, exclude two tables which he introduces, founded on the observations of Adelmann, and Henne.

Out of 49 cases Dr. Adelmann found that

| | | | |
|---|---|---|---|
| In 3 | the length of cord was | | 14 inches. |
| 6 | .. | .. | 15 |
| 12 | .. | .. | 16 |
| 1 | .. | .. | 17 |
| 17 | .. | .. | 18 |
| 4 | .. | .. | 19 |
| 5 | .. | .. | 20 |
| 1 | .. | .. | 21 |

Out of 130 cases Professor Henne found that

| | | | |
|---|---|---|---|
| In 1 | the length of cord was | | 13 inches. |
| 7 | .. | .. | 15 |
| 2 | .. | .. | 16 |
| 4 | .. | .. | 17 |
| 10 | .. | .. | 18 |
| 8 | .. | .. | 19 |
| 16 | .. | .. | 20 |
| 11 | .. | .. | 21 |
| 21 | .. | .. | 22 |
| 9 | .. | .. | 23 |
| 13 | .. | .. | 24 |
| 8 | .. | .. | 25 |
| 3 | .. | .. | 26 |
| 4 | .. | .. | 27 |
| 4 | .. | .. | 28 |
| 1 | .. | .. | 29 |
| 2 | .. | .. | 30 |
| 3 | .. | . | 31 |
| 1 | .. | .. | 32 |
| 2 | .. | .. | 34 |

We now proceed to Dr. Churchill's

* March, 1837.

own investigations. He has had the funis measured in every case attended from the Lying-in Hospital, and the following are the results. Out of 212 cases, he found that—

| | | |
|---|---|---|
| In 6 | the cord measured | 12 Inches. |
| 1 | .. .. | 13 |
| 7 | .. .. | 14 |
| 7 | .. .. | 15 |
| 15 | .. .. | 16 |
| 4 | .. .. | 17 |
| 75 | .. .. | 18 |
| 10 | .. .. | 20 |
| 4 | .. .. | 21 |
| 9 | .. .. | 22 |
| 49 | .. .. | 24 |
| 2 | .. .. | 26 |
| 5 | .. .. | 28 |
| 1 | .. .. | 29 |
| 8 | .. .. | 30 |
| 1 | .. .. | 34 |
| 5 | .. .. | 36 |
| 1 | .. .. | 46 |
| 1 | .. .. | 48 |
| 1 | .. .. | 54 |

Thus, he remarks, out of 391 cases, there occurred six of one foot long, and none under that length. The length which occurred most frequently was eighteen inches, and the next in frequency two feet; so that the estimates of authors are not quite correct. There is but one example in the whole number of a cord exceeding forty-eight inches.

"We may conclude, I think, that cords only ten inches long must be comparatively very rare, (although I have quoted the record of four such,) since not one occurred out of 391 cases. Now, as to the practical effects of these unusually short cords, it should be borne in mind, that (in head presentations) as soon as the breech passes through the lower outlet, all stress upon the cord may be and is taken off by the child's lying with its abdomen close to the vulva; and that the length required is such as will reach from the insertion of the placenta to the vulva, and from the breech of the child, when at the vulva, to its umbilicus. A cord of this length will, it is clear, allow the child to be born safely. What, then, is this length? I have recorded four observations of different authors, in the former part of this paper, of children being delivered without accident, whose cords were only ten inches long. But suppose in these cases that the placentæ were not situated at the most distant point of the uterus, that they were inseited into the side instead of the fundus, we may allow three inches more as the limit of the length necessary, and finally conclude, that a cord of thirteen inches long will always suffice for the delivery of the child, and that one of ten will also, under favourable circumstances. This calculation entirely refutes the notion of delay in labour arising from this cause, unless, at least, the cord be under ten inches, and I am not aware of any example of this being on record."

Out of 190 cases, the cord was twisted round the child's neck in 52. The shortest cord which was coiled round the neck was eighteen inches. This occurred but twice in seventy-five cases. It was never under two feet when coiled twiee round: nor under three, when coiled three times round. It was coiled four times round in one of three feet; and four times in one case of fifty-four inches. Whenever the cord exceeded two feet in length, it was generally round the neck. Deducting the length of the coil in the *shortest* cord, thirteen inches will still be left, which have been already shewn to be sufficient for delivery. The coiling of the cord round the neck seems the consequence of the length of the funis, and the number of coils is proportioned to that length. On the whole then the evil consequences which authors have set down as likely to result from this circumstance, may be deemed imaginary; and the untwisting of the coil which has been much recommended, difficult as it often is, impossible as it sometimes is, in the majority of cases is really unnecessary. The cord should in all cases be drawn down a little, to relieve the stress on it, and that is usually all that is required.

---

### KENT AND CANTERBURY HOSPITAL.

### CASE OF TYPHOID FEVER, ACCOMPANIED BY A DISEASED STATE OF THE BLOOD, AND GIVING RISE, AMONG OTHER LESIONS, TO APOPLEXY OF THE SPLEEN, AS ALSO OF BOTH LUNGS; TO STAINING OF THE ARTERIES; AND THE FORMATION OF SEMI-ORGANIZED CONCRETIONS ON THE PULMONARY VALVES. By JOHN M'DIVITT, M.D. Physician to the Kent and Canterbury Hospital.

We have great pleasure in inserting the report of this interesting case. It has been transmitted to us by Dr. M'Divitt, a gentleman of very high professional attainments, from whom we hope to receive future contributions.

*Case.* On Friday, the 10th of June, a considerable time after the regular hour for admission, a patient was found in the waiting-room of the Kent and Canterbury Hospital. He had been brought from Whitstable, a distance of eight miles, and was so much exhausted, and apparently in so dangerous a state, that he was immediately admitted under the care of my colleague, Dr. Chisholm. It appeared that he had been ill nearly six weeks, and he now presented all the symptoms of typhoid fever. He continued perfectly rational until the period of his death, which took place on the seventh day after entering the hospital.

*Inspection twelve hours after death.*—The body was much emaciated, and there was very considerable cadaverous congestion of back. No petechiæ were visible. The *dura mater* adhered with unusual firmness to the bones of the cranium. The longitudinal sinus was distended with blood, and a great number of large blue veins ramified over the surface of both hemispheres. The arachnoid had lost its transparency, and beneath it was diffused a layer of gelatinous-looking substance, the thickness of which was much increased between the convolutions. The whole substance of the cerebrum and cerebellum, particularly of the former, was highly vascular, presenting, on being cut into, innumerable bloody points, from which oozed out a thin clear red fluid. The ventricles contained each about a drachm and a half of a similar fluid, and between three and four drachms were found in the base of the skull.

The abdomen was now opened, and the vessels of the omentum and intestines were seen beautifully injected with blood of a bright scarlet colour; but as it was becoming dark, the skin and abdominal muscles were drawn together by a suture, and the dissection deferred until half-past seven o'clock on the following morning. On resuming it at this hour, the liver was found to be rather enlarged, but its structure was healthy. The gall-bladder contained about three drachms of green bile. The spleen was much enlarged, exceedingly soft, and permitted its capsule to be separated with the greatest ease.—The septa could hardly be distinguished, the whole appearing one almost uniform mass of soft black blood.—Both kidneys were likewise considerably larger than natural, and felt at the same time soft and flabby. On the surface of the left was discovered a cyst, nearly as large as a marble, containing a transparent melicerous fluid, and sinking deep into the substance of the organ. An incision into the right, divided two similar but smaller cysts, of an irregular oval shape, which were separated from each other merely by condensed cellular substance, while the inner surface of each was deeply furrowed with longitudinal grooves. The stomach contained merely a little mucus. Its lining membrane was slightly thickened, and rather soft, and could be easily separated from the middle coat. Its larger extremity and centre presented several patches (three as large each as a shilling), of punctuated, violet-coloured inflammation, together with, here and there, elevated dark stripes, varying from half an inch to an inch and a half in length, and running in the direction of the pylorus. Near this opening, and between the middle and innermost coats, ramified a small artery distended with scarlet blood, along the trunk and branches of which were observed ten or twelve rounded patches of extravasation, varying in magnitude from the size of a pin's-head, to that of a small pea. The coats of the intestines presented an immense number of beautifully injected vessels of a clear red colour. The intestines themselves were nearly quite empty, and their mucous membrane was healthy throughout. The cæcum was not of larger calibre than the colon. Its middle coat was imperfect, permitting, in two or three places, the lining membrane to protrude between the fibrous fasciculi, pushing out the peritoneal coat, and thus forming thin transparent pouches, one of which, nearly as large as a pigeon's egg, contained a small ball of fæculent matter. The urinary bladder was empty and contracted.

The pulmonary and costal pleuræ on both sides of the chest adhered firmly together. Both lungs appeared very large, and did not subside when the chest was opened, each contained several masses of dark clotted blood, varying from the size of a walnut, to that of a small fist. On cutting them open, some appeared to consist solely of dark grumous blood, while in others, portions of the pulmonary substance half broken down were distinctly visible. Two of the largest masses, situated one in the upper lobe of the right lung, the other partly in this, and partly in the middle one, both of which adhered together round the circumference of the mass—contained in their centre cavities half filled with a dark greenish putrilaginous fluid, such as blood is converted into by putrefaction. The surrounding pulmonary substance was soft and ragged, and in a state of incipient gangrene. Not one of these masses had regularly defined outlines. Portions of them thrown into water were found to float upon the surface. Fully one-sixth part of the posterior and inferior lobe of the right lung had an ash grey colour, varied with reddish streaks, felt exceedingly firm, and presented, when cut into, an imperfectly granulated appearance. Pieces of it thrown into water, sank readily to the bottom. The bronchial ramifications throughout both lungs were much dilated, and contained a small quantity of muco-purulent matter. Their lining membrane was evidently thickened, and presented, here and there, minute violet-coloured spots. The bronchial glands were much enlarged. The pericardium did not contain any fluid. The heart was very soft, and easily lacerated, and, on cutting it open, there oozed out from the divided vessels a quantity of clear red fluid, exactly similar to that which had escaped from the incised brain. The left auricle and ventricle were both empty, and their lining membrane was stained of a bright scarlet colour, which was found to be continued, without the minutest interval, throughout the whole aorta, and its branches, as far as we were permitted to examine them. In the majority of these, the tint was somewhat lighter than in the grand trunk, but in both the internal iliacs it was even deeper, and in each of them was found a small soft clot of dark blood, while all the rest, as well as the aorta itself, were quite empty. The staining, which extended over the whole circumference of the arteries, penetrated into the substance of their middle coat, and could not consequently be removed by sponging, although it was certainly diminished thereby. There was no perceptible thickening or vascular injection of the arterial coats.

The right auricle and ventricle were also empty, and their lining membrane was just perceptibly stained. The aortic, mitral, and tricuspidal valves were natural in structure, as also were those of the pulmonary artery; but to the free surface of each of these last was attached a semi-organized, irregularly nodulated concretion, the central surface of which (if I may so term that opposite to its attached one), presented a sort of cauliflower appearance.—These concretions, or vegetations (for they seemed to partake of the nature of both), nearly filled up the calibre of the artery; and on stretching one of them, so as to separate it partially from the valve, three or four minute and slender vessels were seen to form the bond of its attachment. After these points had been examined, there was found, lying in the ventricle, a smaller mass of vegetations, similar in appearance to those adherent to the valves, from one of which it had evidently been separated in the act of opening the artery, for the point of connexion was still distinctly visible. It presented, when cut into, an appearance resembling that of medullary sarcoma, but it was rather more dense, as well as firmer in its structure. The lining membrane of the pulmonary artery was in every respect perfectly healthy.

Mr. Williams, surgeon, of Whitstable, who had attended the patient previously to his entering the hospital, kindly furnished me with the following particulars.

Bright was 46 years of age. During the last five years of his life he had been employed as a "navigator," namely, in cleaning out and deepening the harbour, an occupation which is very laborious, and which requires those engaged in it to stand up to the knees in water and mud. The men thus employed usually drink very largely of beer, both during, and after the hours of labour. Bright, who had been in the army, where he had acquired habits of intemperance, used frequently to get intoxicated, and in that state to pass the night in the open air. Early in May he was attacked with rigors, followed by feverish heat, and an acute pain in the right side. He gave up his work, and confined himself to bed, but did not call in medical assistance until ten days afterwards, at which time he was found by Mr. Williams labouring under pyrexia, attended with short difficult respiration, cough, scanty yellowish expectoration, and well-marked dulness over right side of chest. He was bled largely from the arm, blisters were applied to the chest, and the system was speedily brought under the influence of mercury. Under this judicious treatment, the symptoms rapidly abated, and the patient, in little more than a week, was able to walk about, and appeared convalescent. He now altogether withdrew himself from medical treatment and control, yet was he not able to resume his employment, but continued to go about the village, drinking rather largely, and taking, as it would appear, very little food. This mode of life he persisted in up to the very day of his admission into the hospital, namely, during a period of nearly three weeks.

*Remarks.* The history of this highly interesting case enables us satisfactorily to account for the very numerous, and varied morbid appearances discovered on dissection. These may be divided into three groups, distinguished from each other by the probable dates at which they severally originated.

In the first group may be classed those which must have existed, and been increasing during a period of several months, perhaps years, viz. the dilatation of the bronchial tubes and thickening of their lining membrane, the adhesions of the pleuræ, the enlargement of the bronchial glands, the renal cysts, and the protrusions of the mucous membrane of the cæcum between the imperfect fasciculi of the middle fibrous tunics.

To the second group belong those morbid appearances, the origin of which is clearly referrible to the attack of illness experienced by the patient between five and six weeks prior to his death. These are, the solidification of a portion of the inferior lobe of the right lung, its ash-grey colour varied with reddish streaks, and also its imperfectly granulated appearance when cut into, being the ordinary results of acute pneumonia.

The last group comprehends a very numerous and varied train of lesions and morbid appearances, all of which must date their origin subsequently to the attack of pneumonia, and some of which could not have existed more than a few days, perhaps hours, before death; they are, the venous congestion of the brain; the injection of its vessels with a clear red fluid; the effusion of a similar fluid into the ventricles, and into the base of the cranium; the opacity of the arachnoid, and the effusion beneath it of a layer of gelatinous-looking substance; the muco-purulent matter in the bronchial tubes; the patches of violet-coloured inflammation in their mucous membrane; the apoplectic state of the lungs; the vegetations growing from the pulmonary valves; the clear continued staining of the lining membrane of the heart and arteries; the striped and punctuated inflammation of the gastric mucous membrane; and the universal injection with a clear red fluid of the vessels of the intestines.

The spleen had, in all probability, been enlarged for a long period antecedent to the patient's death,* but the conversion of its substance into a putrilaginous mass must have taken place subsequently to the attack of pneumonia, and was, no doubt, like several of the other symptoms above enumerated, dependent upon a diseased state of the blood. Was this state the cause, or was it merely a consequence of the typhoid fever under which the patient evidently laboured at the time of his admission? I apprehend that it was neither the one nor the other, but simply an accompaniment, originating in the same causes which produced the fever itself, and constituting in fact one element of the fever. This is a highly interesting question, and as such deserves to be minutely considered. Happily the history of the case throws much light upon it, and enables us to come to a tolerably satisfactory conclusion respecting it. The patient, subject for several years to repeated attacks of bronchitis, engaged in a laborious and unhealthy employment, grossly intemperate in his habits, passing often, while intoxicated, the whole night in the open air, and taking, for days together, scarcely any substantial food—had an attack of pneumonia for which no remedies were employed till after the lapse of ten days. By judicious treatment, however, the disease was in a great measure subdued, but the patient, before his recovery was established, abandoned himself to his old habits, and in consequence speedily became affected with typhoid fever, which went on increasing until the period of his death. Here were a number of causes well calculated to affect at once both the nervous and the sanguineous system, and accordingly there is no ground for supposing that the affection of the one preceded for any considerable length of time that of the other. This is at variance with Dr. Steven's theory of fever, which, I am convinced from numerous other observations, is founded on too partial a view of the phenomena of the disease. I believe also that Dr. Stevens has assigned too much importance to the condition, in fever, of the salts of the blood, and too little to that of the fibrine and albumen, both of which ingredients are very much diminished in fever before the colour of the blood becomes decidedly altered. Indeed I have satisfied myself by numerous experiments, the details of which I may at some future time lay before the profession, that upon the albumen depends, in a great measure, the form, and, to a certain extent, the colour also of the red particles. These, when the albumen is much lessened in quantity, lose their globular shape, break up, and, dissolved in the watery serum, ooze through the coats of the vessel, which are thereby stained of a bright red, more rarely of a violet colour. By many pathologists this staining is considered as the result of inflammation, an error successfully combated by Laennec as also by Bigot and Irousseau. In the present case, its dependence upon a diseased state of the blood was unquestionable, and the nature and appearance of the thin, clear red fluid, which oozed from the divided vessels of the brain and heart, and which manifestly contained little or no albumen, were in perfect accordance with the results of my experiments above alluded to. I would not, however, be understood as asserting that the changes undergone by the blood in fever, depend altogether upon deficiency of albumen: much must be attributed to the impaired influence of the organic system of nerves, from which the coats of the bloodvessels are supplied, and by the agency of which it is that the blood, while circulating, holds in solution a large quantity of

* Since the above was written, I have learned from the friends of the deceased that, several years before his death, he had suffered severely from intermittent fever.

albumen, and maintains the attraction existing between it and the red globules.

I have represented the staining of the arteries as occurring prior to death. That such was the case, is demonstrated by the fact, that the whole circumference of the lining membrane was equally affected, thus shewing that the staining took place while the arteries were filled with blood; for the experiments of Laennec prove that imbibition does not extend beyond the part actually in contact with the blood. It is worthy of remark that the discoloration was deeper in the internal iliacs, in each of which a soft dark clot existed, while all the other arteries were empty.

The apoplectic state of the lungs, like that of the spleen, must be attributed to the diseased condition of the blood, together with diminished tonicity, and even consistence, of the solid fibre, both of which, again, owed their origin to the exhausting agents already enumerated. The undefined margins of the sanguineous masses contained in the lungs, as also the ragged gangrenous appearance of the surrounding pulmonary substance, constituted additional, and undoubted evidence of the extremely sunken state of the vital powers.

The inflammation of the gastric mucous membrane existed, it is probable, only during the very last days of life. I cannot learn that it gave rise to any symptoms indicative of its presence; but the patient, though perfectly conscious, was not in a state to give a just account of his sensations, and as little or nothing was swallowed, the absence of vomiting cannot be a matter of surprise. The small round patches of extravasated blood, existing along the trunk and branches of the distended artery, afforded a beautiful illustration of one very common course of mucous inflammation. Had the patient survived much longer, the numerous patches would, in all probability, have coalesced into one large patch, ulceration or sloughing would have taken place, and perforation ultimately ensued.

When the patient entered the hospital, he presented no visible marks either of difficult breathing or of impeded circulation. The state of the heart, therefore, was not enquired into, and it would now be fruitless to speculate what peculiar modification of sound the valvular concretions gave rise to. The origin, however, and manner of formation of these, may be ascertained with tolerable precision by carefully estimating the date already enumerated. Their degree of organization would indicate an existence of about eight or ten days, but as the natural powers of the system were very low, it is probable that a longer duration ought to be assigned to them. Their loose connexion with the valves, from which, by the rupture of a few slender chords, evidently bloodvessels, they would be readily separated, leaving the valves themselves entire, and in a natural state, proved that they were not primarily the result of vegetative growth, but had their origin simply in a deposition of the fibrine of the blood. Both these modes of production, no doubt, afterwards concurred to bring them to the size which they attained; and as they nearly quite filled the calibre of the vessel, it is manifest that the circulating fluid was greatly diminished in quantity, otherwise very prominent symptoms of impeded circulation must have presented themselves. It is highly probable that the apoplectic state of the lungs favored the fibrinous deposition on the valves, by causing a partial stagnation of blood in the pulmonary artery, and right side of the heart.

*Canterbury, Jan.* 1837.

---

### Statistics of the Rich and Poor.*

Dr. Casper, some short time ago, published some tables, illustrative of the greater value of life amongst the married than amongst the unmarried. These tables Mr. Rickman shewed to be deceptive, and the inferences founded on them he proved to be false. Dr. Casper has lately published some fur-

* Med. Gazette, Feb. 18.

ther statistical calculations on the lives of the rich and poor. But he has calculated from extraordinary data. For the lives of the rich have been estimated from genealogical trees, in which the young and the childless are frequently omitted; and the lives of the poor have been valued from that class which enters hospitals—the healthy, who never go into hospitals, and die of old age at home, being disregarded. When there are such palpable objections to the data, we need not be surprised at the extravagance of the conclusions. That they *are* extravagant will be admitted, when we read that, "compared with the poor, twice as many of the rich attain to the age of 70; three times as many the age of 85; and four times as many the age of 90. In other words, the average duration of the life of the rich is 50 years—of the poor little more than 32 years; a difference of 18 years of life between the prince and the pauper."

The fact is, that the lives of the select class are no doubt better than those of the lower orders. But they are not better to any thing like the amount insisted on by Dr. Casper. It is gratifying to the philanthropist to *know*, that the expectation of life throughout the entire population of England, in the year 1821, had reached that of the select class in the year 1786.

---

### Meath Hospital.

### Clinical Lectures of Dr. Graves.*

Our friend Dr. Graves is a zealous, well-informed, and able physician. A series of his clinical lectures is now in the course of publication in the Medical Gazette. From those lectures we shall select a few points for notice, and we recommend the perusal of them in their native form.

1. *Epidemic Erysipelas.*

This prevailed in the wards of the Infirmary during the months of August, September, and October, 1836. The disease generally attacked the head, commencing in the scalp, or about the nose and cheeks; but in some cases, it appeared first on the nape of the neck, particularly in those patients who had been blistered in that situation during the course of fever. The fever which now prevails seldom abates in less than fourteen or seventeen days; and it was generally about the termination of the febrile excitement, and while convalescence was going on, that the erysipelas appeared. Usually, on the fourth or fifth day of convalescence, a change was observed in the patient, and the erysipelatous attack was ushered in by pyrexia. The erysipelatous inflammation was superficial, and did not affect to any extent the subcutaneous cellular tissue. There was little œdema, and there were no abscesses. There was a good deal of constitutional disturbance and fever, which, in most instances, terminated on the sixth day. In many instances, the erysipelatous redness spread in a perfectly symmetrical manner.

"I believe I was the first who directed attention to the fact, that when erysipelas commences at any point of the mesial line of the body, it is very apt to spread in a symmetrical manner. Thus, in the present instance, the inflammation commenced in the majority of cases about the nose, and then extended in a perfectly symmetrical manner over the forehead and down the neck; or when it appeared first on the nape of the neck, it travelled down between the shoulders with a very remarkable symmetry of extent and outline. Sometimes this precise correspondence did not exist; but I can assert that in more than two-thirds of the cases it was extremely well-marked. It appears, then, that this occurrence is not so very rare as my friend Dr. Johnson supposes. When I first noticed the fact of the occasionally symmetrical spread of erysipelas, he said it was an observation of very little importance, and that it was to be looked upon as a matter of mere curiosity, a phenomenon which a man would not see twice in the course of his life. I have, however, shewn it to

---

* Med. Gazette, *passim.*

many of the students half a dozen times during the last two months."

We suppose we must succumb. We confess that, though we have seen a good deal of erysipelas, we have not had the good fortune to find it so symmetrical. But vision is much sharpened, when we do not merely look *at*, but *for* a thing. It is quite obvious that the fact is not very unfrequent, or our friend Dr. Graves could not shew it so often as he did to his pupils. Still we must regard it as rather a matter of curiosity, than of any thing else.

The treatment was simple, and founded on a consideration of the circumstances under which the disease occurred. No local measures were necessary. Tonics, narcotics, and stimulants were given, and were given at once. With the exception of one case, all were fortunate.

2. *Enemata of Tartar Emetic in the Delirium of Fever.*

Dr. Graves is very partial to the use of the solution of tartar-emetic in the delirium of fever. But sometimes it is very difficult to get the patient to take medicine. What are we to do then? In the first place, the emetic-tartar may be mixed with the patient's drink, which his thirst makes him usually crave. Secondly it may be given in the form of enema.

"The best way of administering it is to dissolve two or three grains of tartar-emetic in four or five ounces of mucilage of starch or isinglass, and inject it with the aid of a long flexible tube, so as to make the contents of the syringe pass high up into the bowel. In this way, you can secure all the good effects of tartarized antimony in overcoming the congestion of the brain, and procuring sleep. In all cases of alarming congestion of the head in fever, I have been long in the habit of using tartar-emetic in this way, if the stomach be deranged, and incapable of bearing it safely; and I can assure you that it is a most fortunate thing to have such a powerful resource in all cases of the kind. I have also not unfrequently given expectorant medicines in the same way, where from the state of the stomach, or the debility of the patient, the ordinary remedies could not be administered by the mouth with sufficient rapidity, or in sufficient quantity to produce the desired effect. In this way I have often given the infusion of ipecacuanha—a remedy of very considerable value, and not sufficiently appreciated by most modern practitioners. I may also remind you that vomiting, and all the benefits derivable from it, may be likewise produced in this way. Now where the stomach is irritable, and yet there exists a necessity for such remedies, it is a very fortunate circumstance to be in possession of a means of employing them without inflicting any injury on the stomach, and thus counterbalancing the good effects of the remedy by the injury done to the stomach. Of course the cases in which these expedients are required are comparatively rare, but the practical physician must be always prepared for such exigencies, and be provided with every means of meeting them."

3. *Thirst of Fever.*

Dr. Graves observes, very truly, that the thirst of fever lies deeper than the surface, and is by no means in the ratio of the dryness of the mouth and fauces. One man, with a moist tongue, will have great thirst, and another, with a dry tongue, will have little.

"There is one curious circumstance connected with the sensation of thirst in inflammatory diseases, which deserves attention. I lately attended a fatal case of metritis after delivery, in consultation with Mr. Hayden and Dr. Ireland. These gentlemen pointed out to me the singular fact, that the patient's thirst was instantly increased to an intolerable degree, by pressure applied to the womb."

It must be owned that an explanation of this fact is not easy. Every body is aware that slightly acidulated beverages assuage thirst. Dr. Graves is of opinion that acidulated bitter infusions are most powerful. A very light infusion of cascarilla, for example, acidulated with muriatic acid, is recommended by him.

4. *Necessity of anticipating Cerebral Symptoms.*

Watch for head symptoms, says Dr. Graves. It is not enough to treat them when they come; they must be seen and met coming.

"You will find in patients who are about to have cerebral symptoms, a degree of restless anxiety, and a higher degree of energy, than accords with their condition; and they either do not sleep at all, or their sleep is broken by startings and incoherent expressions. When you speak to a person in this state, he answers in a perfectly rational manner; he will tell you that he has little or no headache; and were you to be led away by a hasty review of his symptoms, you would be very likely to overlook the state of the brain. If you inquire closely, you will find that he scarcely ever sleeps, or even dozes—that he is irritable, excitable, frequently incoherent, and muttering to himself. Under such circumstances, although there is no remarkable heat of scalp, suffusion of the eye, or headache, I am frequently led to suspect the supervention of cerebral symptoms, particularly about the ninth or tenth day of the fever (for it is generally about this period that cerebral symptoms begin to manifest themselves); and whenever I observe these premonitory indications, I never hesitate in taking proper measures to anticipate the evil."

Dr. G. has the head shaved, blisters it, gives tartar-emetic.

In other cases, the cephalic symptoms are ushered in by drowsiness. The patient seems otherwise well, but he sleeps—sleeps too much. About the ninth or tenth day, he begins to rave, and exhibits undoubted proofs of congestion and excitement of the brain. To be put on our guard is to be armed, in such cases.

Cerebral symptoms are often announced by the state of the respiratory function. Permanent irregularity of breathing and frequent sighing—respiration going on for one or two minutes at one rate, and then for a quarter or half a minute at another rate, bode no good.

---

### BATH CASUALTY HOSPITAL.

**DISSECTION OF THE LIMB, TWENTY-ONE YEARS AFTER THE EXTERNAL ILIAC ARTERY WAS TIED. By G. NORMAN, Esq.**

On the 13th of September, 1813, Mr. Norman tied the right external iliac artery, after Mr. Abernethy's manner, for an aneurysm of the femoral artery, immediately below Poupart's ligament. The name of the patient was Hicks, his age 50 years, and his case was detailed in the 10th volume of the Medico-Chirurgical Transactions. The operation was successful, the disease never returned, and although there were several slight threatenings of apoplexy, and habitual swelling of the feet and legs, the man lived until February, 1834, when he was carried off by cholera. The dissection of the limb is contained in the twentieth volume of the Society's Transactions. It is of course highly interesting, as an addition to our stock of facts.

*Dissection.* "Independently of the appearances in the alimentary canal, characteristic of cholera, there was found some thickening and dilatation of the left ventricle of the heart, with deposition of bony matter in the mitral valve. The arch of the aorta was somewhat dilated, and presented several patches of ossific deposit.

The arteries of the right lower extremity were filled with wax injection from the lower part of the aorta; the injection passing so freely as fully to distend the extreme branches in the foot and elsewhere. The following are the principal results of the subsequent dissection.

The external iliac vein was pervious, and presented nothing calling for remark.

The external iliac artery was obliterated from the bifurcation of the common iliac to a point corresponding nearly to the lower edge of Poupart's ligament, and about one-eighth of an inch above the origins of the epigastric and circumflexa ilii arteries; it there again became pervious, and of equal size with the vessel in the opposite limb. The cord occupying the place of the obliterated vessel was of considerable size; the arterial coats perfectly distinct; and a probe introduced into its centre readily separated the adherent surfaces of the internal coat until it reached a point about an inch above Poupart's ligament, where all traces of the natural textures were lost, and replaced by a dense fibrous bundle.

About four inches below the crural arch, at the outer and posterior part of the femoral artery, was found a mass as large as a small walnut, composed of dense fibro-cellular substance: it involved the first part of the common origin of the profunda and external circumflex arteries, which, though obliterated, could be distinctly traced to the femoral. A portion of the profunda of about two inches was thus circumstanced; no injection having passed into it, and the remaining part of the vessel, which was of small size, being filled by the communication of its branches with other sources of supply.

The collateral circulation had obviously been mainly carried on by the ramifications of the internal iliac artery, which was nearly as large as the femoral. The manner in which it was affected may be described as follows.

1. The ilio-lumbar artery, of very large size, gave several branches to the lumbar plexus of nerves, descending in the substance of the anterior crural nerve as several elongated tortuous vessels communicating at the upper and outer part of the thigh with branches ordinarily derived from the external circumflex, and reaching to the fibro-cellular mass already noticed. Other ramifications of the ilio-lumbar anastomosed with those of the circumflexa ilii, and also by that medium, on the one hand, with the inferior lumbar arteries, on the other, with the external circumflex of the thigh.

2. The obturator artery was proportionally much the largest division of the internal iliac, and with its branches very tortuous. Immediately before its passage through the obturator foramen, it gave off a large branch communicating with the epigastric at a very short distance from its origin, and forming apparently the chief source of supply to the epigastric, which was itself comparatively small. Directly after its emergence at the upper and inner part of the thigh, many of its external ramifications, much enlarged and very tortuous, inosculated freely with corresponding branches of the internal circumflex, also of a large size.

3. The gluteal artery was of average size: some of its anterior branches emerging on the dorsum of the ilium from among the gluteal muscles united with ascending branches derived from the external circumflex of the thigh.

4. The ischiatic artery was enlarged, and communicated tolerably freely with the posterior divisions of the profunda.

5. The internal pudendal artery was also of unusual size, and numerous tortuous twigs, collectively of considerable capacity, derived from its external branches, established a communication between the internal iliac and various branches, chiefly of the internal circumflex at the upper and inner part of the thigh."

We see nothing in this dissection, which calls for remark. It tallies with the experience drawn from the observation of other cases, and with what anatomy would lead us to expect. The re-establishment of the circulation through the agency of the circumflex and epigastric arteries, corroborates the necessity for caution in applying a ligature too near those, or indeed very near any large branch, in the operation for aneurysm, or for wounds.

---

### Guy's Hospital.

### 1. On the Situation and Structure of Malignant Diseases of the Liver.*

Dr. Bright has made some observations on this, by no means an uninteresting subject, in the Guy's Hospital Reports. They may be said to be illustrations of views first exposed in that able patho-

* Guy's Hospital Reports, No. 111.

logist's Gulstonian Lectures, delivered in 1833.

He stated it then as his opinion, and that opinion he continues to hold, that probably the cellular membrane connecting parts is the chief seat of the "malignant" changes, which assume a variety of forms, owing to the structure of the particular organ, and owing likewise to the particular modifications to which the morbid action itself is prone.

"We are naturally led to suppose," he remarks, "that morbid changes so marked should constantly originate in some particular tissue: and if this be the case, I should be inclined to assign the locality of its invasion to the cellular membrane connecting the firm parts of other structures. The investigations of Dr. Hodgkin upon the subject of the anatomical structure of malignant growths serve to shew that, in many cases at least, a regular cellular arrangement is assumed by them; which he compares to combinations of reflected membranes, and ascribes to a peculiar power of growth arising from the diseased action. It has seemed to me probable, when considering the structures which he has so beautifully demonstrated, that the disease is first set up in the natural meshes of the cellular membrane, which is universally distributed; that the ordinary serous secretion of those minute cavities becomes changed to that more viscid character which approaches closely to the appearance of mucus, or the more firm opaque substance of the cerebriform cancer, or the sanguineous secretion of fungus hæmatodes, or the ink-like deposit of melanosis;—that this state of diseased action communicating from cell to cell, the natural cells become enlarged, and their parietes thickened, gradually involving in the disease whatever structure they encounter. Thus, in parts where the cellular membrane is loose and diffused, as in the ovaries, in which there is likewise a natural tendency to form cysts, from the peculiar functions of the part, these cysts become greatly distended: where, on the contrary, the surrounding parts are less yielding, they are compressed; and where they meet with other organs, these become involved, the disease intimately pervading their structure, possibly still introduced through the medium of the cellular membrane, whose natural, almost aerial secretion, becomes changed by contiguity into the same morbid secretion, whether it be mucous, cerebriform, sanguinolent, or melanotic: certain it is, that occasionally, in examining tumors of a much milder character — even common steatomatous tumors—the same structure, or one very much resembling it, may be traced in them, as in tumors of malignant growth; but the lobules of which they are composed give an impression that the cellular adipose membrane has, by a more partial and confined action, been secreting a fatty matter, instead of the peculiar deposits which mark the direful march of malignant disease.

'When we examine even ovarian cysts, where the circumstances allow the peculiarities of growth pointed out so beautifully by Dr. Hodgkin to display themselves in all their force and variety, we often find the mucous secretion not held within decided cysts, but lodged in cavities, whose crescentic margins seem to resemble the orifices which communicate between the cells of the cellular tissue, magnified and thickened by pressure and disease: but, at the same time, I am inclined to believe, that while much of the structure depends for its form on the natural meshes of the cellular membrane, in many cases additional cells are formed by newly-generated membranes; and that under other circumstances, even without the formation of such secreting cells, a peculiar vascular arrangement takes place, from which the malignant matter is rapidly deposited by numerous newly-formed vessels.'"

1. Dr. Bright proceeds to explain the variety of the malignant growths.—Thus, in one case, a glutinous secretion occupies and distends the cellular meshes, and displays little tendency to become organized. We see this peculiar form of disease in the structure about the pylorus, in the mamma, in the glands near Glisson's capsule, in the liver, in the kidney, and particularly in the ovaries. Dr. Bright conjectures that the pressure exercised by the effusion tends to diminish or obliterate the communications between the cells, and thus to account for the limitation of the morbid products.

2. In other cases, the cells are occupied with a matter of a more opaque colour and cream-like consistence as well as more capable of organization. This is the true "medullary sarcoma." The looseness of the deposit inadequately supports the vessels which shoot into it, and they are apt in consequence to give way. All the varieties of fungus hæmatodes are produced in this way.

"This medullary mass has within itself a vascular structure, exerting a kind of propulsive force: and though, while in very cellular parts, as those connected with the ovaries, we find it protruding itself into successive cells, which it distends, in imitation of the mucous and glairy secretion to which I have referred, yet in more solid parts, as the liver, we rather find it pushing the neighbouring parts aside, and involving and corroding the cellular structure, which quickly ceases to present a regular appearance of rounded cells, and, as the mass becomes softened and broken down, can only be traced in the form of ragged fibres pervading the medullary growth."

3. Melanosis appears to be very analogous to the medullary and hæmatode alteration.

4. While the growth of the genuine scirrhus is slower, and its deposit of a different kind, its seat appears to be the same as that of the preceding. The scirrhous deposition extends along the meshes of the cellular membrane, and pursuing diverging or radiating directions, it may assume more or less of a spherical form.

Such is the substance of the views of Dr. Bright. We are not sure that there is any thing peculiarly novel in them, for it has long been an opinion with some that the cellular membrane is the nidus of the malignant depositions.

Dr. Bright illustrates his views by three cases and two plates. The first case was one of soft malignant tubera of the liver—the second was an instance of hard malignant tubera of the liver—and the third was an example of the small diffused scirrhous tubera of the liver. Both the cases and the plates are apposite, but it does not appear necessary for us to notice them further. A sketch of the pathological views they illustrate seems to be all that is requisite.

### II. Case of Hydatids Discharged from the Lungs.*

Ann Smith, aged 15, admitted into the clinical ward, under the care of Dr. Cholmeley, Dec. 16, 1835.

Her countenance was flushed; she felt pain about the sternum and scrobiculus cordis, upon taking a deep inspiration: her tongue was covered with a loose white fur; and her pulse was 120, full and jerking, but compressible.

The anterior part of the chest was healthily resonant, upon percussion, on the left side; as also on the right, above the mamma; but below this there was great dulness, extending to the inferior part of the axilla; and it was also observed posteriorly upon the same side, from the spine of the scapula downwards, where a flattening of the parietes was observed: the posterior part of the chest sounded well. Cavernous respiration, with shrill gurgling, and very distinct pectoriloquy, were heard just below the right mamma, and over a large space posteriorly. Mucous and sonorous rattle were heard over the greater part of the left lung. The impulse of the heart was great and diffused.

She had generally enjoyed good health, with the exception of some pain in the right side, occurring at distant periods, and attended with slight cough. She followed her occupation, as a servant, till eighteen weeks previously to her admission, when she was seized with pain in the chest, attended with expectoration and febrile disturbance, for which she was placed under medical care. As these symptoms diminished,

* Ibid.

diarrhœa and profuse perspiration, most excessive in the morning, supervened, and continued till her entrance into the hospital.

Cupping, leeching, blistering, and demulcent medicines were employed. The pain subsided, but the other symptoms, conjoined with much action of the heart, continued.

"*Jan.* 1. Last night she complained to the nurse of being much worse; and at five o'clock this morning felt herself, suddenly, nearly suffocated. She said, she thought something had given way in her chest: and coughed up continually, in large quantity, a dark brown offensive muco-purulent matter, some portions of which were mixed with blood, and contained cysts, which were flattened and compressed, and had the character of hydatids: countenance anxious and depressed. She is bathed in profuse perspiration: bronchial wheezings audible at a distance: no pain in the chest: pulse 130: jerking, soft.—Pergat.

2. She has continued to expectorate matter of a lighter-brown colour, partly tinged with yellow, and containing cysts which are now distinctly recognised as hydatids (some of which are preserved in the Museum): the sputa in the last twenty-four hours amount to about two pints: she complains of no pain in the chest or region of the liver: tongue thickly coated: three loose motions: some fulness in the right hypochondrium.

Mucous and sonorous rattle, and moist crepitation, are to be heard above the inferior angle of the right scapula: below, there are cavernous respiration, loud gurgling, and a very clear resonance of the voice: these are heard also, but in less degree, at the anterior and inferior part of the right side, and at the lower part of the axilla: there is also gurgling in the subclavicular region, but no pectoriloquy. The bronchial rattles in the left lung continue.

Mist. Cretæ c̄ Conf. aromat. bis die. —Omitte medicamenta alia.

4. Matter expectorated much the same as to character, resembling eggs beaten up: the presence of bile in it is shewn by the chemical tests: the cysts, which are not entire, may be inflated: aspect improved: tongue less furred: pulse 120."

The sputa continued bilious, while the dejections were white. On the 12th of January, a cyst was found in the expectoration, and a part of one was again, and for the last time, discovered on the 18th of March. On the 20th of January, the expectoration began to diminish greatly in quantity, and on the 2nd of February, it was scanty and colourless. On the 28th of Jan. we are told that respiration may now be heard below the right mamma, and the dulness, on percussion, is diminished. The pectoriloquy is increased in intensity, but is much less clear.

On the 22nd of February, the report states, that there is still some gurgling below the right scapula, when she coughs; and at that time there is distinct pectoriloquy. Above there is good respiration, and normal resonance of the voice. The whole of the left lung is apparently healthy.

On the 1st of March, her health was nearly re-established, and she was free from cough. Two inches below the inferior angle of the scapula, and at the lower part of the axilla, on the right side, dulness still existed, respiration was heard at a distance, and there was ægophony.

On the 6th of March, there was a relapse, the cough and yellowish expectoration returning. By the 25th, however, the expectoration had ceased, and on the 2nd of April, she was discharged, looking very well. On the 10th of May she was again seen. She had grown remarkably fat. The crepitant rattle was heard when she coughed, but over a limited space.

This is, pathologically speaking, an interesting case. There can be little question that the hydatids formed in the liver excited inflammation in that organ, in the diaphragm, and inflammation and adhesion in the contiguous lung—that suppuration in the liver and lung, and ulceration through the diaphragm ensued—that the hepato-pulmonary cavity containing pus and hydatids opened into a bronchus—and finally that, by this means, the cysts and the matter were both expectorated, and the patient cured. The stethoscopic signs were both distinct and valuable.

### III. Wound of the Ulnar Artery—Hæmorrhage Restrained by Pressure on the Radial and Ulnar.

A boy, aged 10, fell down and wounded the ulnar artery with a flint, just above the left wrist joint. The accident was immediately followed by a considerable arterial bleeding, which was restrained by pressure on the wound. The hæmorrhage, however, occurred upon various occasions, apparently whenever the bandages became loosened; so that the boy's health began to be affected by the repeated bleedings: and he was therefore brought to Guy's Hospital on the 4th of June, five weeks after the accident had occurred. At this period, a wound of considerable size extended obliquely, about three inches in length, from the styloid process of the ulna, upwards and outwards, towards the radius; being filled with large glassy granulations, presenting what is vulgarly called the appearance of proud flesh. So soon as pressure was taken from this wound, the bleeding immediately recurred; but not from any one point, so as to lead the surgeon to a bleeding vessel, but the blood seemed to flow from the whole surface.

Pressure, therefore, was again applied, by the dresser for the week; which was, however, required to be so strong, in order to stop the bleeding, that all sensation in the hand was soon lost: and it was therefore quickly removed, when bleeding as quickly returned. A tourniquet was then applied on the brachial artery, and Mr. Cooper was sent for.

Upon examination, it was found that pressure upon either the ulnar or radial artery singly did not check the hæmorrhage; but that a very slight pressure upon both, continued but for a short time, succeeded. Mr. Cooper, therefore, ordered a piece of cork to be placed upon the radial and ulnar arteries, about two inches above the wound, and to be confined in that situation by means of strips of adhesive plaster, with a degree of pressure not sufficient to prevent the flow of blood through them, but merely to diminish its volume: the hand, at the same time, was raised to an angle of 30° from the horizontal plane of his body, and directed to be kept cool, with evaporating lotion. This treatment most perfectly succeeded; and in the latter end of June he was discharged, perfectly well.

---

### On the reputed Cure of Hydrocele by Puncture.

Perhaps there are few operations which have excited a greater stir of late, than the important one of puncturing a hydrocele with a needle. The removal of the superior maxilla does not seem to have excited half as much attention. Nobody appears anxious to lay claim to that, but surgeons are on the qui vive to enjoy the merit of proposing or performing the other.

Every body knows how easy it is to get up a crowd in London. A man has only to stand still and look at the moon with great intensity, and fifty other people will stand still and do the same. The principle of imitation is a very strong one in all animals, and more especially in man.

There is something very charming in being a discoverer. Like writing, it signifies little what you find, so that you find out something.

> A book's a book, altho' there's nothing in't.

These truisms have been remarkably borne out by some late publications on the operation for hydrocele to which we have alluded. If any thing might be supposed to be established, it was that merely puncturing a hydrocele would not cure it. It was because puncturing would not and did not cure it, that the operations by incision, the seton, and injection were invented. All this seems very clear, to any one acquainted with the history of the treatment of hydrocele. Nay, oddly enough, analogy appears to lead to the same conclusion.

Tapping for example, is not found to cure ascites, nor ovarian dropsy, at least it is not generally found to do so.

If tapping a hydrocele with a trocar will not cure it, it is not, à priori, likely that tapping it with a needle will. Reasoning does not seem to lead to such a supposition. But reasoning, is in medicine proverbially fallacious, and, in this instance, it would appear to have turned out so.

A short time ago, Mr. Lewis, brought before the public, his mode of treating hydrocele, by puncturing the tunica vaginalis with a needle. He made a single puncture. On withdrawing the needle, a drop or two of fluid oozed through the aperture, but gradually the remainder of the fluid, escaped from the cavity of the tunica vaginalis into the cellular membrane of the scrotum, and was there absorbed. Mr. Lewis proposed this as a palliative mode of treating hydrocele, but hinted that it might become a radical one.

The mode of operation is this.

"The operator should grasp that part of the tumour formed by the dropsy as much as possible apart from the testicle, so as to be able to give, by pressure, that tenseness to the skin which is necessary for the easy penetration of the instrument. This he should continue to do while he withdraws the needle, to which, in its passage out, he should give some rotatory motions *on its axis*. The removal of the needle will leave one drop of the liquid from the tunica vaginalis on the skin. The appearance of a drop of the fluid at the top of the little wound is the test of the operation being well performed. It is a demonstration that the bag has been perforated, and that its contents can find their way out; and should it not take place, another puncture must be made. Care must be taken to press in the needle perpendicularly, and to rotate it well after it has entered the tunica vaginalis; otherwise the opening will not admit of the passage of the fluid from it.

After the operation, a compress moistened with a discutient lotion, may be kept on the scrotum, and the patient may walk about or take rest, as best may suit him."

Not long after Mr. Lewis, Mr. King wrote a paper highly laudatory of Mr. Lewis's method. That paper displays what must certainly be deemed rather sanguine expectations. After describing the mode of puncturation, Mr. King remarks:—

"The phenomena which present themselves in a few hours are very remarkable:—The swelling begins to be less circumscribed, and to lose its tenseness, and the cellular tissue of the scrotum becomes gradually more and more infiltrated with the fluid which before distended the tunica vaginalis, and which in the space of from twenty-four or forty-eight hours, will have entirely changed place. In five or six days this infiltration disappears, and the patient is cured. These effects explain the process by which a cure is obtained."

Mr. King goes on to explain the rationale of the cure.

"It appears that the fluid very gradually oozes from the serous sac into the cellular tissue, making its way from one cell to another, till at last anasarca of the scrotum is substituted for the circumscribed dropsy of the tunica vaginalis. At the same time that the cellular tissue is filling, the serous sac gradually returns upon itself—an effect of the utmost importance as regards the ultimate result; for it appears that by this gradual contraction it loses more or less completely the condition necessary for the reproduction of the fluid, and yet remains inclosed, so as to exercise its proper functions. Probably the contraction is not wholly active—is not alone the result of the contractility of tissue belonging to the tunica vaginalis combined with the action of the cremaster muscle. We believe the pressure of the fluid may have something to do with it; for be it observed, the liquid, once out of the sac, presses on its exterior, and becomes an obstacle to its future distention. Each drop, as it oozes from the bag, becomes a force which helps to squeeze out that which is to follow: and thus has Mr.

Lewis achieved a victory perhaps never before gained, by making the product of a disease battle against the disease itself. Once distributed in the cellular tissue, the fluid is quickly removed by the absorbents."

If Mr. King's ideas are strictly correct, there is a very great virtue in the gradual contraction of the tunica vaginalis. No doubt this is likely to assist a cure, but, so far as we can see, its merits are not such as to establish a very important difference, between the puncture by a needle, and that by a trocar. If mere puncturing could generally effect a cure, we doubt whether the plus or minus of time occupied in the exit of the fluid, would be of very great moment.

After Mr. King came Mr. Travers. This gentleman vindicated his claim to the invention, and described his mode of performing the operation. It is this.

"*My mode* of proceeding is to put the scrotum on the stretch, in front of the testis, by embracing it with the extended thumb and fore finger of the left hand; and placing the patient opposite a window on which the sun falls, or at all events a strong light, I command the view of the transparent bag so perfectly as to avoid veins and any point of accidental adhesion or thickening, which is always marked by a corresponding opacity. The punctures are made in a perpendicular direction and in quick succession, about equidistant from each other, while the tunic is kept tense by graduated compression. Of several cases thus treated, *some* have remained cured; others *apparently cured*, have *relapsed* after a fortnight or three weeks, and one after three months; but I do not consider this a fair criterion of the value of the practice, because from the shape and size of the acupuncture needles chiefly used in these cases, the points on which I believe the success of the operation mainly depends were not accomplished.—These are, a freer collapse of the sac by the removal of a sufficient portion of the fluid which the trochar puncture ensures, and the more complete diffusion of the remainder into the surrounding cremaster and cellular tunics, obtained by the multiplication of the punctures, while the tension of the sac is preserved.

On the third day the fluid is absorbed, and the two sides of the scrotum are uniform: indeed this is sometimes the case on the second day; but if the punctures are so small as not at once sensibly to reduce the bulk of the swelling, a single drop only exuding at each orifice, the reduction is generally much slower, or even fails altogether. Now every body knows that the drawing off of the fluid of a hydrocele through a canula is almost invariably followed by its reaccumulation, and it is not uncommon for elderly men, who are timid, or averse to injection, to call upon their surgeon to have the tunica vaginalis relieved of its contents once in a year or in two years."

Mr. Travers goes on to point out the bizarrerie of laying down the method in which a cure takes place, when it is by no means certain that there *is* a cure at all. Mr. Travers thinks, as most men of experience will, that the comparative merits of puncturation with a needle and with a trocar are not determined, and that the *radical* merits of the former are still more equivocal.

Mr. Lewis, in his reply to Mr. Travers, affirmed that many punctures are "not only useless but dangerous," a position in which we totally differ from him—and that they are "less likely" to succeed than a single puncture, a conclusion which is exceedingly dubious.

To this point then the matter has been brought. Mr. Lewis proposes one puncture with a needle as a palliative cure of hydrocele, and conjectures that it may be made a radical one.—Mr. Lewis considers himself the "inventor," because he first performed the operation between two and three years ago. Mr. Travers recommends several punctures,but speaks, having often operated, very doubtingly of the success of the plan.

In this stage of the business Mr. Keate made his début.

"While I do not mean," says Mr.

Keate, " to intimate any doubt of the same ideas having occurred to each of these gentlemen without any knowledge of the other's theory or practice, or of any previous operation of the kind, I trust it will not be offensive to either of them if I assure you that the plan and the practice have been known and acted on for very many years by myself, and I dare say by others. By one other person I know it was performed I dare say *twenty years ago*, namely, by a friend of mine, who for some years practised as a physician in this town, and is now living in retirement in the country. This gentleman performed the operation on *himself*, as he was nervous about the injection, and fancied, as he said, that if he could convert ascites into anasarca, absorption from the cellular structure might cure the malady; and in his own case it was perfectly successful.

At his suggestion I tried it frequently, both at the hospital and in private practice; sometimes successfully, but more frequently the collection of fluid in the sac returned, and I generally found the patients impatient of the numerous punctures, and of the time required for the absorption. I remember talking to Sir Astley Cooper on the subject, and, as far as my recollection serves me, the plan appeared not to be new to him."

We can bear Mr. Keate out in two points; 1, we saw him try the plan six years ago, and we tried it then in three or four instances, and in two or three others subsequently, ourselves; 2, we have not happened to see the operation cure the hydrocele in any one.

On the whole we think it is pretty clear, that, as a mode of treatment generally to be depended on, for the radical cure of hydrocele, a single puncture, or many punctures may be looked on as a mere " matter of moonshine." Now and then a cure will take place, but too rarely and too uncertainly, to admit of any dependence being placed on it.

It must be regarded as essentially a palliative proceeding.

As such, its supposed advantages over puncturing with a trochar are:—1, its occasioning less pain;—2, its more effectual nature from the slower contraction of the tunica vaginalis.

As such, the advantages of puncturing with a trochar over it are:—1, *that* it is more speedy;—2, *that* it is less painful than many punctures, and probably not more painful than one. After all, it is doubtful whether the slow contraction of the tunic is of service,—and taking all in all, weighing the expedition of a trochar puncture, with the tardiness, and the uncertainty of a needle puncture, our own opinion is decidedly against the latter, even as a palliative mode of treatment.

While we say this, we do not mean to affirm that there are no cases in which it might be judiciously employed. But we are sure they are the exception, not the rule.

---

### SALISBURY INFIRMARY.

### CASE OF RECOVERY FROM THE INSENSIBILITY OF INTOXICATION, BY THE PERFORMANCE OF TRACHEOTOMY. By S. SAMPSON, Esq.

This case is contained in the last volume of the Medico-Chirurgical Transactions. It reflects much credit on Mr. Sampson, of Salisbury, and is physiologically, as well as practically, interesting.

*Case.* " Abraham Harris, aged 31, was brought to my house on the 31st of March last, in a state of complete insensibility from intoxication, the pupils being largely dilated, the breathing stertorous, and all voluntary motion having been lost for at least four hours before I saw him. The account given by those who came with him was, that he had attended a convivial meeting in the course of the day, at which he had drunk freely both of beer and brandy; his companions admitted that he had taken more than a pint of the latter, but it has since been ascertained that his glass was repeatedly filled up, without his knowledge, with white brandy, instead of water, so that it is impossible to calculate what quantity of spirit he had actually taken.

I immediately used the stomach pump, and drew off between three and four pints of fluid, a great part of which appeared to consist of brandy; after which, tepid water with ipecacuanha diffused in it was several times injected into the stomach, and after a while withdrawn again. with a view to excite vomiting, and thus rouse the energies of the brain. Finding, however, that these means failed, a strong solution of salt in water, and afterwards the sulphate of zinc, were repeatedly tried, without any better result; but he became, if possible, more comatose, the countenance turgid, breathing more and more difficult; the pulse grew fainter, and was at last scarcely perceptible; at the same time the whole surface of the body was cold and clammy, and he was insensible to every kind of stimulus. As he was some miles from his home, I had him removed to the Infirmary, and called a consultation of the other medical attendants, who arrived in the course of half an hour; but as, in addition to the above symptoms, he had lost the power of swallowing, and every appearance indicated the rapid approach of death, nothing was ordered for him but a turpentine injection, there being no ground to justify a reasonable hope of recovery.

At this period it occurred to me whilst standing by his bed-side, that the comatose state in which he lay might not arise from apoplexy, but from torpor of the brain, in consequence of that organ being supplied with blood not duly oxygenated; for the shrill tone and extreme difficulty of respiration shewed the existence of collapse of the glottis, and imperfect transmission of air into the lungs, which might be accounted for by a paralysed state of the eighth pair of nerves and recurrent branches. With this view of the case I again appealed to my colleagues, and strongly urged that a trial should be given to the operation of tracheotomy; for I could not but hope that if mechanical respiration were carried on for a time, the blood might regain its proper stimulant properties, and restore the energies of the brain and nervous system. Upon their consenting to give him this chance, the operation was performed, without loss of time, by Mr. Andrews, under whose care, as surgeon for the week, the patient was now placed.

The trachea was no sooner opened than the distention of the veins about the head and neck subsided, the violent efforts of the extra-respiratory muscles ceased, and in about half an hour regular and easy respiration through the wound was completely established; at the same time the pupils became slightly sensible to the stimulus of light, and the pulse returned to the wrist. The immediate result of the operation being thus far satisfactory, nothing remained to be done but to give directions for the frequent removal of the mucus which appeared at the wound, and to keep the surfaces of the incision asunder until the integuments and muscular layers had become agglutinated to each other; this latter object was effected by means of a piece of strong spring wire, with a bow at each end of it, which, being introduced in a bent state, was allowed to expand, and the opening in the trachea was thus prevented from being covered by the muscles, even during the efforts of deglutition.

He continued perfectly quiet during the night, but had no return of consciousness until the following morning, when he gave us to understand, by signs, that he suffered from headache and soreness at the pit of the stomach; there was a tendency to sickness, and the tongue was coated with a peculiar whiteness, as if rubbed over with chalk. Moderate purgatives, followed by mild alkaline medicines, soon removed these symptoms, and a few leeches were applied to the throat, for the purpose of checking too high a degree of inflammation; after which no further treatment was required; but the wound being healed in about three weeks, he was discharged cured, and has continued up to this time in the enjoyment of perfect health."

We think there can be little doubt that the patient was dying of asphyxia, the result of the depressing influence of alkohol upon the brain and nerves. The opening made in the trachea facilitated respiration, by establishing an aperture, not muscular and regulated by nervous influence like the glottis. In such a case, were the symptoms still more urgent, and tracheotomy ineffectual, artificial respiration should, of course, be kept up. The effects of alkohol are transitory, and the view with which Mr. Sampson advised the operation is perfectly philosophical, and was, happily, successful.

---

### MARY-LE-BONE INFIRMARY.

#### CASE OF RECOVERY FROM THE INFLUENCE OF OPIUM BY ARTIFICIAL RESPIRATION. By C. IRVING SMITH, Esq.

This case may be considered a valuable pendent to the one immediately preceding. It too is contained in the last volume of the Medico-Chirurgical Transactions.

*Case.* Jane H., aged 25, a stout young woman, was admitted into the St. Mary-le-bone Infirmary, on the 20th of July, 1828.

" At six in the morning, she was observed by one who slept in the same room, to swallow something from a cup, the remains of which was opium. She shortly after became insensible. At 10, A. M., she was brought in. At this time, her extremities were cold and livid; the lips and face of a dark lead colour, the pulse fluttering, and scarcely perceptible at the wrist. Respiring three or four times in a minute, with sighing.

The stomach pump was applied, and that organ was carefully washed out, first with water, then with dilute acetic acid; after which, small quantities of ammonia were injected, combined with brandy. The spasm of the œsophagus was so great, on attempting to introduce the tube of the stomach pump, as nearly to destroy it.

The patient was now (11, A. M.) evidently worse than at the time of her admission; so much so, that many doubted if she still lived. The hair was next entirely cut off from the whole head, and the head placed over the edge of the bed, several buckets of cold water were poured in rapid succession over it; the nostrils being at the same time stimulated with volatile alkali. The pulse now intermitted much at the wrist, at one time being 70 or 80 beats in the minute, and the next sinking as low as 7 or 8. Respiration had now nearly ceased.

The scalp was now rubbed with the liq. ammoniæ until the entire of it was vesicated.

11½, A. M., it was determined, as the only remaining chance, to try the effects of artificial respiration; there being at this time no pulse whatever at the wrist, and only a slight irregular action at the heart, indicative that life was not quite extinct.

The mouth and one nostril being closed, a pair of common bellows was applied to the other nostril, and the chest was in that way inflated, and alternately emptied by pressure on the chest and sides. This was continued for an hour, without intermission, at which time the heart seemed to be rallying, but if left to itself, it rapidly sunk again. An ounce of the ol. terebinth. was injected into the rectum. Bottles of hot water were applied to the chest, and sinapisms to the feet and legs.

The same treatment was continued till 2, P. M., with only very slight intermission. She was at this time so evidently rallying, that it was deemed safe to leave her.

She was again seen at 3, P. M., when she was found relapsing into her former state. Recourse was again immediately had to artificial respiration, which was continued till 5, P. M., at which time it was suspended, on account of the pulse having become regular, and her having made some slight attempts to move; and shewing evidence of pain on being pinched.

At 12, P. M., she became slightly sensible, and swallowed a little tea. She became eventually well; and has since married.

It was curious to see the effects of artificial respiration in this case. The livid colour of the face and extremities, rapidly giving way to a more florid hue, on every inflation, and again as rapidly returning to its original colour, on being left for a few minutes."

---

### Midwifery in the French Lying-in Hospitals.

Dr. Lilburn has communicated to the Medical Gazette an account of what he saw in Rouen and Paris. The following extract gives no flattering account of the state of midwifery in the provinces of France.

"Rouen.—The Hôtel Dieu, in this city, contains 600 beds. One ward is appropriated for women during their confinement, who are admitted by the physician, and kept as long as he thinks judicious (generally one month.) The woman, when in labour, is taken into a small room, and there delivered on her back, with the head and shoulders a good deal raised, in the presence of the pupils. Immediately after delivery she is carried to a room appropriated for her reception, and the infants to one adjoining. In that for the latter their little beds are arranged round the room with canopies; they are dressed in swaddling clothes, confined all but their arms. One woman had been delivered the day before I arrived with forceps (Baudeloque's). She had lacerated perineum, and laceration through the vagina into the rectum. The Interne, who accompanied me round the hospital, and very politely explained the whole progress of the case to me (bringing me specimens of the instruments then in use), said, that the accoucheur, in employing the forceps, although a very powerful man, and exerting the whole of his strength, had the perspiration running down his face, and was more than an hour in extracting. The ergot had been previously given, and the patient bled largely.

I remarked that the mischief might probably have been avoided by the patient lying on her left side, and by the use of the curved forceps, invented by Dr. Osborne, and modified since his time, as there was no deformity of the pelvis.

He told me that perforation of the head of the child, when necessary, was effected with a long sharp-pointed bistoury, and the brain removed principally by the operator's fingers!"

---

### Clinical Cases of Morbid Growths from the Bones of the Face, and Observations on their Nature and Removal.

#### 1. Case of Exotosis of the Bones of the Face. By Mr. Morgan. (Guy's Hosp. Reports, No. II.)

L. Shiel, aged 24, admitted into Guy's Hospital, Aug. 1, 1835, with a tumor on the right side of the face, which had commenced, when he was fifteen years of age, in the right nostril, and had subsequently gradually increased.

The whole right side of the face was occupied with the morbid growth, the right nostril being enormously expanded and closed by the enlargement of the tumor, which, from its size, completely concealed the eye on that side, and extended downwards into the mouth, being there connected with the palatine and alveolar processes of the right superior maxillary bone; projecting also forwards, so as to press the lip beyond the teeth, to the extent of two inches. The bones apparently implicated in the disease were the ossa nasi, superior maxillary bone, vomer, and the inferior turbinated and malar bones.

A lithographic sketch represents the tumor; the surface was irregular; the more prominent parts being situated under the right orbit, and opposite to the ala of the nose. They were slightly discoloured by inflammation; but the reddened surface of those projections indicated no approach whatever to the change in the condition of the vascular system, which evinces so frequently the local existence of a truly malignant disease. The patient complained of no pain—the general health seemed good

—and, although he was much emaciated, that was probably from want of food.

The tumor being, throughout its whole extent, almost uniformly firm, and of a bony hardness, and sufficient time having elapsed, from its first formation, to give rise to malignant softening if the disposition to it existed, Mr. Morgan concluded, and his opinion was supported by that of his colleagues, that the disease was simple exostosis. He determined, then, to operate, which he did on the 6th of November.

"Desirous of ascertaining, in the first instance, the true nature of the disease, I made a crucial incision, between the most prominent points, over the right nostril; and, by means of a trephine, removed a portion of the tumor, which proved to be true exostosis.

A semilunar incision was then made, extending over the nostril, from the internal angle of the right eye to the centre of the upper lip. A similar incision was made on the outer side, commencing at the external angle of the eye, and joining with the other, at the lip. The integuments were then dissected from around the tumor, and a metacarpal saw was used for its removal; and as it was of a spongy texture, it offered little resistance to that instrument. No great quantity of blood was lost during the operation, the exostosis not being very vascular; and it was only found necessary to secure one vessel, a superficial branch of the transverse facial. All further disposition to hæmorrhage was easily restrained by pressure.

After the tumor had been thus removed, the integuments were brought together by the uninterrupted suture; a dossil of lint was placed over the wound, and confined by adhesive plaster; and over all, a light bread-and-water poultice was applied.

The patient bore the operation with great fortitude; and said afterwards, that he suffered but little pain, excepting when the first incision was made.

A section of the tumor proved that it was composed of an outer hard thin shell of bone, completely enclosing a morbid mass of spongy cancellated structure, devoid of all appearance of carcinomatous or fungoid disease."

There is nothing to detail in the progress of the case. The wound suppurated gently, and in April, 1836, when the Report was published, all discharge from the face had almost entirely ceased, hardly any exfoliation of bone had taken place, and the general health of the patient was restored.

### II. Case of a Large Bony Tumor of the Face, removed by Spontaneous Separation. With Observations on some of the Functions of the Soft Palate and Pharynx. By Mr. Hilton.

The following case is interesting on physiological accounts. The separation of the tumor permitted the examination of the pharynx and palate, during respiration, deglutition, and speech.

*Case.* T. Moore, aged 36, in good general health, stated that "About 23 years ago, a little pimple, like a wart, appeared just under my left eye, close to my nose. I scratched off the head of this pimple; which formed a scab; and ever since there has been a growth from under that spot."

The tumor grew slowly, until it occasioned extreme disfigurement of the face. The turbinated and cellular apparatus on the left side of the nose were destroyed; the septum nasi was pushed towards the right side, so as nearly to obliterate the right nostril; and the left orbit was thrust outwards.

In a short time, the growth of the tumor displaced the inner wall of the orbit; and the globe of the eye, being then subjected to pressure, became the seat of most excruciating pain, though vision was very little impaired: the symptoms however continued; until, about 17 years ago, the globe, yielding to the pressure, burst, and gave exit to its fluid contents. The cicatrix of the cornea is still obvious, pointing out the part of the globe which gave way.

This yielding of the globe was attended with very little bleeding, but it produced almost complete relief from pain. He has subsequently been subject to occasional neuralgic pains, following the distribution of the first and second divisions of the fifth nerve. The tumor did not extend backwards to the pharynx. His voice became nasal and that was all.

About six years ago, the tumor was observed to be somewhat loosened, and detaching itself by ulcerative absorption from the surrounding soft parts, the integuments being destroyed by its pressure forwards. This process was accompanied by copious suppuration, and occasionally by profuse arterial hæmorrhage, proceeding not from the vessels of the tumor itself, but from those of the adjacent structures about the situation of its origin, near the internal angle of the orbit. About eighteen months ago, several small irregular portions of bone came away, and these were followed, when the bands of skin ulcerated, by the separation of the whole mass. A very large chasm was, of course, the consequence. It was bounded *below* by the nasal surface of the hard palate and the floor of the left antrum: *above*, by the left frontal sinus and left half of the cribriform plate of the æthmoid bone: *internally*, by the septum nasi, which presented a general concave surface, with a small opening through it, at the lower part, communicating with the right nostril; and *externally*, by the left orbit. *Posteriorly*, it opened into the pharynx.

The roof, the outer wall, and part of the inner are now covered with granulations, more or less abundant, with here and there small portions of bone, denuded but not presenting any malignant character.

The tumor weighs fourteen ounces and three quarters. Its density is remarkable, the specific gravity being 1.80. Its greatest circumference measures rather more than eleven inches, and its least nine inches. The external surface is irregularly nodulated, and an uneven concavity exists at the posterior part. A section presents a very hard polished surface resembling ivory; and exhibiting lines, to the number of fifty, arranged in concentric curves, enlarging as they are traced from the posterior part. Dr. Hodgkin, who examined the tumor with accuracy, and details the results of that examination with scientific precision, does not deem it of a malignant character—a conclusion borne out by the history of the case, as well as by the general features of the morbid growth. By chemical analysis, the tumor was found to contain less animal matter, more earthy phosphates, and less carbonate of lime than ordinary human bone.

By a report dated July, 1836, the patient would appear to continue in good health, and the chasm of the face has a little diminished. Judicious surgical attentions may no doubt materially diminish the deformity.

We observed that the case was possessed of some physiological interest. The opening in the face exposes in their perfect state and natural position the pharynx, and the hard and soft palate, and exhibits, of course, the actions of the soft parts in respiration, deglutition, and articulation. We will extract a few of the more prominent circumstances.

1. *Respiration.* The opening in the face represents an exaggerated nasal aperture.

*a.* During quiet and unconstrained breathing, *with the lips closed* so that the air must pass through the nose and opening, there is not any perceptible movement upwards of the soft palate; nor does the pharynx advance. The latter remains perfectly still; and the former, we may conclude, as it disappears in the descending direction, is closely adapted to the back part of the mouth, so as to leave, for the air passing to and from the larynx, a free and spacious canal.

When *the lips are slightly separated*, so that the respiration is in part carried on through the mouth, the soft palate is seen to be carried, or rather, perhaps, drawn upwards and backwards at each inspiration, and at each expiration again to decline; the pharynx continuing almost at rest, but having a slight disposition to advance.

Upon taking a full inspiration through the mouth, the palate is directed more completely upwards and backwards, and adapts itself to the advancing pharynx.

This adaptation remains until the expiration has nearly terminated: but it should be remarked, that the sides of the pharynx do not, even in this case, approximate so much as during deglutition.

From these observations we may conclude, that during hurried or forced inspirations and expirations made with the mouth widely opened, nearly all, if not the whole of the air which enters and escapes from the pharynx and larynx is excluded from the nose, and takes its course through the mouth."

*b.* In smelling there is no perceptible movement of the palate nor of the walls of the pharynx.

"If the odoriferous particles employed be very *pungent*, a different result takes place; as was observed in the following experiment:—A piece of wood, previously dipped into liquor ammoniæ, was held within the large cavity in the face. The patient did not smell it, nor did it make him sneeze; although it created a sensation of heat in the nose. This sensation of heat, gradually extending into the pharynx, its parietes involuntarily advanced, and the palate was raised so as to preclude the further descent of the ammoniacal vapour.

The same co-aptations between the palate and pharynx occur if the scent be in any way *disagreeable*."

*c.* "In holding the breath, as it is termed, or forcibly retaining the air in the chest, either with the lips closed and the tongue depressed; or with the tongue raised and adapted to the concave surface of the hard palate, and the lips separated; or, lastly, with the superior and posterior portion of the tongue applied, by great voluntary effort, to the concave commencement of the soft palate, the lower jaw being depressed and carrying with it the genial portions of the genio-hyo-glossi muscles; the pharynx advances, and the palate is much raised, presenting a convex surface upwards, undulating, with a somewhat tremulous motion, upon the subjacent column of air."

Mr. Hilton observes that the closure of the glottis alone, can retain the air within the chest but for a very short time, and that the assistance of the soft palate, as well as of the tongue and lips and jaws, is highly useful.

*d.* Sneezing consists of inspiration through the mouth when the palate is raised, and of expiration through the nose, when the palate is depressed. But if sneezing happens many times in quick succession, so that the palate has scarcely time after its elevation, during the oral inspiration, to descend to the back of the mouth before the air returns from the chest, the palate is then seen violently agitated by the air in its ascending direction, being at that time obliquely placed with respect to the expired air, so that a portion of the air passes through the nose, and a portion through the mouth, giving the coarse tearing sensation to the palate, and flapping motion of the lips, experienced in the imperfect attempt to sneeze.

2. *Deglutition.* When the mouth is open to receive the food, the palate is raised, but not so completely as in full oral inspirations: the sides of the pharynx also approximate, but not so closely as on drawing the breath inwards: the posterior part of the pharynx advances but slightly. Directly the food, either solid or fluid, is placed in the mouth, the palate descends, and continues, during the detention of the food, closely adjusted to the back part of the mouth, the pharynx remaining perfectly quiet. These conditions are to be observed during the process of mastication, performed with or without food in the mouth. Extreme lateral movements of the jaw encroach upon the pharynx but very slightly; and in the ordinary lateral motion, there is no difference to be observed in its capacity. Immediately antecedent to that part of the process of deglutition occurring in the passage of the food between the fauces and pharynx, or when the food is passing backwards over the upper opening of the larynx, the palate is carried *completely* upwards and backwards, and the pharynx advances, the sides of which more especially approximate. At this time the thyroid cartilage rises. These movements of the larynx and palate occur nearly simultaneously; those of the palate having but a momentary precedence. The palate and pharynx being now nicely adjusted, their common surface presents, from above, a hollow cone, in consequence of the partial descent of the palate, but so closely is their adaptation maintained during the deglutition, that not the slightest portion of the passing substance is perceived above the palate. At that moment the pharynx is indeed divided into two distinct cavities; one at the upper part common to the nose and pharynx, the other open to all the apertures below the palate. As the fluid or food passes into the lower part of the pharynx, the upper portion recedes or retires from the palate; the soft palate falls; and, last in this succession of events, the larynx, as is indicated by the motion of the thyroid cartilage, descends.

3. *Articulation.*—On this head, Mr. Hilton's observations are imperfect, and it is not necessary to go into them. On the whole the case is interesting, and Mr. Hilton deserves the thanks of physiologists, for seizing the opportunity of ascertaining, by actual observation, the changes which occur in the soft palate and pharynx during the performance of deglutition and of respiration. The mechanism is both beautiful and perfect, and though reasoning has anticipated much of what Mr. Hilton has seen, yet philosophy is always under obligations to those who add positive facts to her stores.

We had intended to have entered fully on the subject of the tumors of the superior maxillary bones, and on the operations required or performed for them. But the want of space compels us to pause, and to defer till our next Number the consideration of this subject.

---

Clinical Observations of the late M. Dupuytren on Contusion, Compression, and Displacement of the Heart.

The following fragments from this illustrious surgeon's lectures, are extracted from a monthly journal, just established by our intelligent friend, Dr. Bureaud Riofrey. This gentleman is a French physician now resident in London, whose liberality of feeling and cultivated understanding are highly estimated by all who have the pleasure of his acquaintance. His aim, a laudable one, is to lay before the profession of this country the valuable facts and memoirs published on the Continent, and to cement those generous bonds of brotherhood which unite the lovers of science and philosophy, whatever their country or their clime. Country, indeed, and clime are distinctions which, in this sense, philosophy does not admit, and which the natives of France, to their honour be it spoken, have often openly disavowed. Desgenettes and Larrey extended to the British soldier, whose bayonet had been pointed at them, the same zeal, the same science, and the same humanity, with which they assuaged the wounds of their own comrades. Such conduct was worthy of them and the profession, a profession which, above all others, is consecrated to the service of the human species.

We would willingly aid Dr. Riofrey in his attempt to draw more closely the ties which unite the children of France and England. In arts, if not in arms, in civilization and refinement and all that adorns and ennobles humanity, the two first nations of this Globe—scarcely separated by nature—sprung from common parents—and calculated, if not intended, to diffuse by their union the blessings of order, of liberty, and of science—he must be devoid of every spark of enthusiasm and of generosity, who would not hail their alliance as a happy epoch in their history.

The Journal before us, established with this view, and conducted in this spirit, is termed the Continental and British Medical Review. We wish it all success, and we shall direct the attention of our readers, from time to time, to its contents.

The fragments from the Lectures of M. Dupuytren on some of the lesions of the heart, are not unworthy of attention. They embrace conditions or affections of that organ not generally noticed by pathological writers.

1. *Traumatic Compression of the Heart.* J. L. Petit, some time since remarked, that if the fractures of the sternum were ill united, there resulted a very uncomfortable palpitation of the heart, owing to the pressure of this organ by the displaced fragment. In a case of this kind, spitting of blood has also been occasioned. Dupuytren, who had deeply studied the diseases of the heart, found these observations of Petit's very correct, and had various opportunities of ascertaining the fact. Nevertheless he had seen these palpitations gradually disappear, under the influence of lowering treatment, sometimes merely by the assistance of nature. The possibility of this latter phenomenon would be easily admitted, if we recall to mind that bony fragments introduced in the chest, cease to injure the thoracic organs, as they are blunted by the repeated impulsions of the organs with which they are in contact.

The compression of the heart may also be caused by the effusion of the blood in the pericardium, which may cause instant death. (Scarpa.) Or else by the introduction of a foreign agent which remains in the neighbouring parts. The following extraordinary case was related by Dupuytren in one of his lectures:—

An officer in the army labouring under great distress of mind had resolved to destroy himself: to accomplish this purpose he thrust two long hair pins in the region of the heart. Serious thoracic accidents ensued, but the cause was unknown, for the patient kept his secret; different remedies were administered, but there was no abatement of the sufferings.

The pins were so deeply pushed in that there even remained no signs of their introduction. The primitive symptoms disappeared, but the pain continued, and the palpitations were very severe. The ensuing year this wretched man put an end to his existence in another manner. On a post mortem examination, two long hair pins were discovered in the interior of the chest near the heart, irritating this organ, in the same manner as the fragment of the sternum to which we have already alluded.

The traumatic compression may sometimes be carried so far as to cause sudden death by suffocation. In such a case pressure on the heart prevents the circulation of the blood, and of course respiration is immediately stopped.

The following cases are related in illustration of the preceding observations:—

*Case.* A young man playing at nine pins fell on a stone, and died instantly; and at the post mortem examination was found a fracture of the sternum with intro-pressure of the inferior fragment, which weighed on the heart.—(Duvernay.)

*Case.* An immense stone fell on a labourer who was working in a quarry; his chest was crushed, and he died of compression of the heart.

2. *Contusion of the Heart.* It is much more difficult to ascertain the simple contusion of the heart, for its effects are nearly similar to the preceding lesion. When compression is carried to such an extent as to cause suffocation, it must necessarily contuse the organ on which it acts. Dupuytren said that the contusion of the heart produced lypothymy more or less dangerous, consecutive carditis, and palpitations more or less durable, and more or less difficult to cure.

It is almost unnecessary to add what should be the treatment of compression and traumatic contusion of the heart. To attack the cause if possible, and by weakening medicine, prevent too strong a phlogistic reaction is the best, and the only principle on which the practitioner can act.

It must be owned that the observations on contusion of the heart are not so satisfactory as might be wished.

3. *Displacement of the Heart.* "The permanent displacement of the heart through traumatic causes has hitherto scarcely been noticed. The following is however a remarkable example of this case :—

A young mechanic of one and twenty, was caught in the wheel of a machine, and horribly mutilated,—two of the lower ribs on the left side, and the fifth, sixth, and seventh on the right side—the humerus and the right clavicle were fractured. Excruciating pain and pulsations similar to those caused by a foreign agent, were felt on the right side. The heart had passed to the right of the sternum. At the end of a month, the patient was better, but his respiration was oppressed, and the pulsations were felt on the right side. The slightest emotions increased their violence. Cold suddenly striking the hands or the chest, brought the patient to a state of suffocation. His health was very bad during three years : vomiting came on after every repast, and the winter was generally marked by numerous pulmonary congestions, with increase of the other symptoms, and principally great pain in the right side.

Digitalis was prescribed in doses of ten grains every night, after which dyspnœa, and the palpitations were diminished ; the pulse, which was at 120 and 130, fell to 80. The chest was deformed, the lower part was dilated more than an inch, the shoulder was depressed. The left side resounds on percussion ; the sound is very clear between the fifth and seventh ribs : normal situation of the heart. The noise of its contractions or pulsations is not heard in all the left side ; respiration feeble. The sound from the upper part of the right lung is clear. Under the fifth rib a great sensitiveness and pain, no respiratory noise, no bronchic sound. The heart is felt, in the right mammary region, between the sixth and seventh rib, an inch below the sternum. Its impulsion precedes, at stated intervals, the pulse of the radial artery.

After so precise an examination, the displacement of the heart could not be doubted. The normal situation of the heart had been ascertained before the accident happened. There are many well known cases of displacement of the heart, arising slowly from the development of a tumor, either solid or liquid, in the chest, but nothing of the kind has yet been published resulting from traumatic violence."

---

### Opinions of a French Surgeon on the State of the Ophthalmic Hospitals of London, and of the Profession in this Country.*

Dr. Bourjot St. Hilaire has lately been in London. He writes of it like a gentleman and a man of science. Too many Continental physicians and surgeons have paid a flying visit to this metropolis, and knowing nothing of our language or our manners, have confidently pronounced opinions, too often ridiculously false, upon our practice. Such is not the case with Dr. Bourjot St. Hilaire. We will quote a passage.

" The hospitals (ophthalmic) are not so large in London as in Paris, and this is an advantage ; for they are more dispersed, and more within the reach of a patient's friends—and, according to the lower class of people, they appear less like hospitals. The wards are lofty, well lighted, and do not hold more than fourteen beds ; there is a wide space between them, and there are many little utensils which allow the sufferer to fancy himself *at home*. All the furniture is well painted, and therefore easily washed ; the floors are of timber, and cleansed every morning—there are no offensive smells, so common in France, particularly at the *Hôtel Dieu*, and which is a powerful cause of insalubrity.

In each ward there is a fire-place and a good fire, so that there is always boiling-water to make the tea, which is brought to the patients by their friends ; and this beverage serves as diaphoretic, stimulus, diuretic, aperient, for each and all cases.

In fine weather there are no bed curtains, excepting for the patients who have undergone operations ; besides

* Continental and British Med. Rev.

which, at the foot of the bed, there is a screen. It would be very advisable to introduce this custom in our hospitals, particularly in the ophthalmic wards: as it protects the patients from currents of air far better than curtains."

M. Bourjot appears to have arrived at a pretty accurate estimate of the state of the profession in this country, and, we suppose, in all. He had, possibly, heard that the faculty in England were rolling in wealth, and abounding in honour. A very brief acquaintance with their physiognomy convinced him that they were neither one nor the other.

"Let it not be supposed that the practice of medicine in London, or in any part of England, has prompt and pleasing results for the young practitioner. The difficulties are the same in England as in France, in London as in Paris; the same jealousy, the same rivality—in fact this fine profession does not, either in England, France, or Italy, give the advantages that might rationally be expected from a great advance of money; an education prolonged to an advanced period of life, a continual exercise of the intelligence: and medicine, destined to play so great a part in society, is frequently the most laborious, and less lucrative profession."

These observations of Dr. St. Hilaire's corroborate some which we have made on the subject of medical education in a former part of this Journal. The profession, taking it en masse, is any thing but well off. A ruinous competition prevails in its ranks, and the majority of its members must look forward to a life of labour, with little prospect of accumulating such a competence as may maintain themselves in age, or support those dear to them whom they leave behind.

But it must not be supposed that there exists any occult cause for this unfortunate result. That is just what might be expected to flow from a system of medical policy which encourages rather than represses the influx into our ranks—a policy the effects of which will be felt more keenly every day—a policy which some insane radical reformers are clamorous to extend.

One has only to look on a knot of medical men collected on any occasion in a large city, to be satisfied that "the world is not with them," or at least with too many of them. The "shocking bad hat," the rusty black coat and breeches of the same description assiduously brushed, the muddy complexion and care-worn countenance, and the lean and hungry ensemble, tell, alas, in words that cannot be misconstrued, that toil and anxiety and disappointment are the lot of many a noble mind—and that men whose feelings and whose education have qualified them to fill a respectable station in society, are compelled by necessity to eke out a scanty livelihood, from the unwilling payments of insolent shopkeepers and artizans.

From the cry that has been got up against the "Pures," a person who knew nothing of the matter would suppose, that these were the depositaries of the wealth and the success of the profession. There never was more arrant stuff. The majority of the "Pures" are well known not to make "salt to their porridge." A man who should now go into practice as a "Pure" must either have very superior talents, a very strong introduction, or some private fortune. Ninety-nine out of the hundred of young "Pures" make nothing at all by their practice, if that term should be facetiously applied to what they do. If you wish to see a young Pure, you do not look at the carriages, "cabs," or tilburies that are passing, you do not dream of seeing him emerge from a door whose knocker is tied up, but you go to the board-room of a hospital, or to the operating theatre, or dissecting room. There they are in shoals, neatly dressed, gentlemanly, quiet young men, with a strong expression of expectation in the countenance, and a consciousness of merit not yet drawn out. They chat, stroll round the wards, and warm their nether ends at the board-room fire until two or three o'clock, when one of them suddenly recollecting a very urgent appointment, the others as suddenly remember the same thing, and all hurry, they alone know where.

The few "Pures" who do succeed, enjoy, no doubt, what prizes the profession of medicine has to offer. But they *are* a few, and, as at the Bar, there are more called than chosen. If a young man commences as a general practitioner, industry and good conduct will pretty certainly obtain him a subsistence. But a "Pure" is certain of no such thing, and many an one, after waiting for some tedious years, with scarce a fee per annum, has retired in disgust from the profession, and preferred the scanty stipend of a curate, or the precarious chances of the law. Too many instances of this sort have occurred amongst our own contemporaries.

---

### Norfolk and Norwich Hospital.

### Report of Surgical Patients admitted in the Year 1834. By J. Goodwin Johnson, Esq. Assistant Surgeon.

This Report is contained in the last volume of the Provincial Society's Transactions. It is short, and so must be our notice of it.

It appears that this hospital contains upwards of a hundred beds, and is officered by three physicians, three surgeons, an assistant-surgeon, and a resident apothecary.

The number of patients admitted between the 1st of January and the 31st of December 1834, amounted to 658. Of these the in-patients were 469—the out-patients 189—and the casualties amounted to 247, being more than a third of the whole number. The deaths during the year were 20, the number among the males being 17, that among the females 3.

The causes of death were as follows:—Two from *burns*, viz. a child under 10, and a woman of 80;—three from *stone*, viz. 1, a man who came in with a stone of large size, and inflamed bladder; he died in a few days: 2, a man aged 63, having had symptoms for four years, was lithotrized, and died of inflamed bladder, &c.—3, a child 3 years old, having had symptoms for one year, was cut, and died in a few days;—two from *fracture of the skull;*—one from psoas abscess;—one from general cachexia:—one after amputation, on account of a diseased knee-joint;—one from cut-throat;—one from fracture of the spine;—one from malignant disease of the testis;—one from gangrene of the foot;—one from sloughing of the urethra;—one from disease of the lungs, supervening on psoriasis;—one from compound fracture of the arm and thigh;—one from a catheter, broken in the bladder;—one from disease of the lungs, supervening on fracture of the arm;—one from a cause not stated.

Of the 658 patients admitted, there were—

| | |
|---|---|
| Cured | 448 |
| Relieved | 53 |
| Died | 20 |
| Went away without leave | 14 |
| Discharged at their own request | 14 |
| Not likely to receive benefit | 8 |
| No account given | 51 |
| Remain under treatment | 50 |
| | 658 |

Of the 51 of whom no account is given, a very large proportion consisted of trivial cases, which were evidently cured, but did not attend to be discharged.

The greatest number of patients admitted were between the ages of 10 and 30. Nearly a half of the whole number, 293, were between those ages.

We may now mention some circumstances and cases, which deserve a special notice.

1. Several of the cases of posterior curvature of the spine were successfully treated, by rest on a firm mattress, the adaptation of a wooden splint, well padded, to the projecting vertebræ, and stretching, which was effected by fixing the head to the upper end of the bedstead, and making careful and graduated extension of the hips and legs.

2. A vesico-vaginal fistula, arising from a long and protracted labour, was not benefited by caustics, cautery, or ligatures.

3. Ten male patients were admitted with calculi: six were cut, of whom one died, and five were cured: two refused to remain in the house; one was a man of seventy, with a very large stone, having had symptoms for ten years, and was not considered a proper subject for the operation; and one died shortly after his admission, from inflamed bladder and diseased kidney.

4. A portion, more than five inches long, of a catheter, made of soft metal, somewhat firmer than a metallic bougie, was broken off in the urethra and escaped into the bladder of an old man of 80; it was seized by Weiss's forceps and extracted. He died six days afterwards, being sixteen from the accident.

*Examinatio cadaveris.*—The intestines were firmly bound to the bladder by adhesions of long standing; the coats of the bladder were considerably thickened, and its cavity much contracted; the mucous membrane was vascular and dark; the right lobe of the prostate gland was enlarged, and the left much more so; the third lobe, which formed a projection into the cavity of the bladder, was soft, spongy, and vascular, and had been perforated by some instrument. The neck of the bladder was found uninjured by the operation, but the lining membrane of the urethra was lacerated in several places. The urine in the bladder was mixed with muco-purulent matter.

5. *Cases of fracture of the skull.*—These are interesting.

CASE 1. *Compound Fracture of the Skull—Bad consequences from removing a Foreign Body.*

James Becket, aged 16, admitted in the afternoon of January the 12th. He came from a distance of four or five miles in a cart, and got out and walked into the hospital without assistance. On examination, the breech-pin of a gun, which had burst in firing, was found to have penetrated the frontal bone, just in the situation of the superior longitudinal sinus. He gave an account of his accident, answered questions well, and was not at all confused. The surgeon who had charge of him, proceeded to remove the breech-pin, which was firmly impacted, and afterwards took away three or four portions of bone, one of which contained part of the ridge to which the superior longitudinal sinus is attached. The aperture in the cranium was now nearly an ich in diameter. The dura mater was wounded, and a portion of cerebrum escaped on the removal of the foreign body. The wound was lightly dressed. *Very soon* afterwards he became restless and uneasy; complained of pain, and was much confused. Pulse 50, and intermitting; his pupils rather dilated. Half an hour after the operation he became comatose, and his breathing stertorous; and he had slight convulsive twitchings of his *left arm.*—Ten o'clock p. m. Pulse rapid and small; pupils of natural size; passes his urine involuntarily; breathes heavily; the dressings were thrust up by a protrusion at the wound.

13th, morning.—Had been insensible all night; his left arm moving convulsively; the right side of his body paralytic; pulse 150; pupils of natural size, but inactive; respiration hurried; unable to swallow.—Evening. The left side is now become paralytic; tumour of brain more prominent.—14th. Died at ten this morning, without convulsions.

*Examinatio cadaveris, Jan. 14th.*—The tumour protruding through the aperture in the cranium, seemed to be composed of dense layers of coagulated blood and cerebral substance, of a dark colour, sloughing and disorganizing; and the brain in immediate contiguity with the hernia, presented a somewhat similar appearance. The tumour arose from the right anterior lobe of the cerebrum, near the superior longitudinal sinus, which had been wounded on the side next the hernia, and a large quantity of coagulated blood was found both above and beneath the dura mater in this situation. The brain, in general, was pale and bloodless.

The surgeon who had the charge of the patient, communicates his opinion on the case, in the shape of the following note.

"This boy walked to his ward on arriving at the hospital; was quite sensible, and continued so, without any symptoms of compression, *until after the breech-pin was removed,* when he became, in a short time, comatose with stertorous breathing and dilated pupils, and died in less than forty-eight hours. Death, in this case, ensued from pressure being removed: the brain being left not only *uncompressed,* but unsupported, at the seat of injury, so that some of it escaped through the wound, and a hernia cerebri was quickly formed from the impetus of the circulation. Thus death was expedited by the injured brain protruding so as to interrupt, more completely, its functions. Had I allowed the breech-pin to remain, life would, I believe, have been prolonged, and have terminated, in the course of some days, from inflammation of the membranes of the brain. How are we to reconcile this case with the present doctrines of our greatest authorities, in regard to compression of the brain? The symptoms which quickly shewed themselves on the removal of pressure, in the above case, were precisely those enumerated as the *symptoms of compression.* We can only say, that such a train of *symptoms* are the effect of interruption to the cerebral functions, and that compression is *one,* but *not the only cause* capable of producing these symptoms."

We must confess that this hypothetical explanation of the case is far from satisfactory. The following objections to it readily present themselves.

*a.* In cases where the brain is wounded, and portions of it escape, or protrude in the form of hernia cerebri, such symptoms as occurred in this instance are not common. On the contrary, in cases of hernia cerebri, symptoms of compression have repeatedly been induced by the actual application of pressure to the fungus.

*b.* The superior longitudinal sinus was wounded, coagulated blood was mixed in layers with the cerebral substance, and a large quantity of coagulum was found both above and beneath the dura mater. Here was an abundant source of compression, and this, with the disorganized condition of the cerebrum, is sufficient to explain the symptoms in a satisfactory way.

So far as this case goes, it supports the propriety of the recommendation given by many of the best practical surgeons, not to meddle, if possible, with foreign bodies impacted in the brain. But the treatment of these cases is uncertain, after all, and it is difficult to determine what is really the best practice. Perhaps, in this as in other instances, it is well to adopt the cautious maxim, of not doing any thing, when what we do *may* be injurious.

Case 2. *Fracture of the basis—consecutive effusion of blood.*

Martha Ward, aged 68, admitted at 10 P. M. on the 18th of September. She had fallen down three or four stairs at twelve o'clock at noon; was not aware how long she remained prostrate, but had got up without assistance; she was quite sensible, and had described her fall to a medical student, who visited her soon after, and who thought her case trivial. At three o'clock she became comatose, and her breathing was stertorous; and she remained in this state until six o'clock, when a surgeon saw her, and bled her to twenty ounces. On admission, she was insensible, and her breathing stertorous; her pulse small and frequent; her skin pallid and cold; there was loss of power in the left eye-lid, the pupil of that eye being contracted and fixed, whilst that of the right eye was dilated and sluggish. She was continually carrying her right hand to her face, but scarcely moved her left arm; she moved both her legs when tickled on the soles of the feet, but the left less actively than the right; her urine and fæces passed involuntarily; there was a very small wound behind the right ear, which, however, did not communicate with the bone: no fracture was felt. She became gradually worse, and died at seven o'clock in the morning of the 20th.

*Examinatio cadaveris, Sept. 20th, at half-past eleven.*—There was a fracture of the right parietal bone near the posterior inferior angle, a portion of bone, an inch long by half an inch wide, being insulated and loose, but not depressed.

A fissure extended from this spot, *upwards*, along the lambdoidal suture for an inch and a half; and to the same extent across the parietal bone and *downwards*, dividing the petrous portion of the temporal bone, and the mastoid process, and proceeding through the tympanum and eustachian tube, to the fissura lacera, at the base. On removing the calvarium, a large coagulum, weighing three ounces, was found between the dura mater and cranium; it was one inch and a half thick, and produced a corresponding indentation of the brain. The blood proceeded from a branch of the arteria meningea media, about two inches from the external wound; the brain and plexus choroides were pale; there was about half an ounce of fluid in the ventricles.

No doubt the symptoms were those of concussion in the first instance. The coma, stertor, paralysis which followed, resulted from the extravasated blood. That would seem to have begun to flow in any quantity, about three hours after the occurrence of the accident.

CASE 3.—*Compound Fracture and Depression of the Skull—no "Symptoms"—Operation—Recovery.*

It is in accordance with the present canons of surgery to operate in such a case as this. There is reason to believe that the operation is advisable in cases of compound fracture and depression, unattended with symptoms, while it is not to be recommended, in cases of simple fracture and depression without symptoms. This distinction was first insisted on by Sir Astley Cooper. The present case, so far as it goes, bears out the precept.

James Pye, aged 11, admitted December the 15th; there was a wound, almost parallel with the sagittal suture, over the left parietal bone, which was bare, and a triangular portion of it depressed, the depression being about twice the thickness of the cranium, and gradually diminishing. He was quite sensible, and answered questions readily and correctly; pupils active; pulse regular. The wound was enlarged, and a portion of the parietal bone, overlapping the edge of the depressed bone, was removed by Hey's saw. The depressed bone was then removed, the outer table being broken into three pieces, whilst the inner table remained whole. About six ounces of blood were lost during the operation. On the 18th, the pulse was quick, and he answered rather incoherently; but, after that, there were no further bad symptoms.

---

SKETCH OF THE HOSPITALS OF THE BRITISH AUXILIARY LEGION IN SPAIN.

For this we are indebted to a young naval surgeon of much ability, who occasionally visited the hospitals of the Legion, and had opportunities of observing the manner in which they are conducted. He does justice to the exertions of the medical officers, who deserve great credit for what they have effected, in spite of difficulties of every description. Amongst those officers, we may signalize Mr. Alcock, whose experience, gained at the siege of Oporto, and in many bloody fights in Portugal, has been happily applied to the mitigation of the sufferings of his brave comrades in Spain.

"There is a painful interest attached to all the circumstances connected with the present contest in Spain; to none perhaps more so than to those of the unfortunate British Auxiliary Legion, and its very able, zealous, and laborious medical staff. From their first landing in that distracted and apparently doomed country, the medical officers of the Legion, but especially those on the Staff, have had their hands fully employed, in a variety of practice such as can rarely, if ever, fall to the lot of men engaged in more private life. The resources of those gentlemen have been taxed to the uttermost; their health and strength have in too many instances given way under a combination of adverse circumstances. Death destroyed many, and many more have been compelled to retire with the bankrupt estate of a broken constitution—an empty purse—and the already *dishonored* bills of the Spanish paymaster-general. A

band of determined men remain to abide the issue of the war—and continue their assistance to ameliorate its horrors. It is to be hoped that some of them will hereafter furnish their professional brethren in this country with a medical history of the British Auxiliary Legion—such a work, drawn up with care and with a moderate degree of talent, would supply one of the most conclusive arguments against such wild and futile enterprises as that in which they have been engaged. The disastrous consequences of their sojourn in Vittoria—the ravages of typhus there, at Bilboa, at Santander, and at San Sebastian—the cart-loads of killed and wounded brought in, from time to time, to the military hospitals—and the deplorable state to which hundreds are already reduced—would furnish abundant materials for such a work. The multitude of interesting facts afforded by nearly two years military practice at the seat of war itself, might render it of incalculable value to the military branches of the profession. And the melancholy exhibition it would make of the ignorance, vanity, conceit, and dogged obstinacy of the Spanish and other executives in resisting the humane efforts of their medical attachés to give efficiency to their department, might teach a salutary lesson to those who will only learn lessons of humanity, or even common-sense, when taught them in the hearing of that public on whose breath they feed and live.

The writer of these observations was in no wise connected with the Legion. It was not his lot to serve in that body—and to any credit that is due to the medical branches of it, he has no claim. It is, and ever will be, matter of regret to him, that the very harassing nature of his own employment left him few opportunities of improving his acquaintance with the labours of his professional brethren in that, in many respects, unfortunate corps.

But the want of ability and energy which appears to characterize the executive department of the Legion, is not visible in the medical, or, as it is contemptuously designated, the Civil department. And the provision made for the comfortable reception and treatment of the sick and wounded soldiers is creditable to all concerned. At Santander and San Sebastian, between which places the different regiments are divided, very respectable hospitals have grown up for the reception of casualties, &c. and the general order, cleanliness, and attention paid to the patients, might be transferred with advantage to some of our more costly establishments nearer home.

There is but one hospital at Santander, pleasantly and healthily situated on an eminence, overlooking the town and harbour. The interior is far cleaner than the exterior, a somewhat strange anomaly in either public or private buildings in a Spanish town. The beds well supplied with bedding—and arranged at convenient distances in three parallels. The attendance good. And the patients quiet, orderly, and cheerful, for the most part, notwithstanding their sufferings. Most of the cases there were convalescent, at the time of the writer's visit—and none of particular interest. A very virulent form of itch was prevalent among some of the inmates, who were separated from their companions in consequence, and, half naked, set to rub one another from head to foot with the ung. sulph. comp.—They were deplorable objects—and I never before saw the disease assume so fearful a type.

At San Sebastian there are two hospitals (English), San Telmo and La Lonja. The former is appropriated to the reception of surgical cases principally—the latter was chiefly filled with cases of fever. Typhus, happily, milder than usual, was, at the time of inspecting it (November 1836), prevailing in the town, and La Lonja was accordingly excessively crowded. Most of the patients lay two in a bed, many of them had not so much as a shirt to cover them. The effluvia were intolerable, and, it must be confessed, that the contrast between it and San Telmo was very broad, and by no means favorable to the mode of conducting it.

San Telmo occupies an extensive site under the castle hill, and on the eastern shore of the isthmus connecting that promontory with the main land. Formerly a monastery, one may be sure the "holy fathers," who chose it for their residence, paid especial attention to all the advantages of sight and sense, before deciding to domiciliate there.—As a surgical hospital it may be said to have grown out of the action of the 5th of May, when the edifice was desecrated to the purposes of a Spanish barrack. The wounded, after that action, were obliged to be distributed in different buildings, from the want of a suitable edifice for their general reception—and the inconvenience and excessive labour resulting from such a state of things led to the ejection of the sound and healthy soldiery from this convent, and its subsequent conversion into a hospital for the wounded.

It has extensive accommodations—is well attended—and evidently the subject of admirable and consistent discipline. There are, besides offices, storehouses, and a tolerably well-stocked dispensary, five long, lofty, and airy wards, each of which is said to be a "division," and has been taken advantage of for the better classification of patients according to their respective cases.

The *first* division contained only *slight* cases, in which general designation are included diseases of the eye and eyelids, flesh wounds and their sequelæ, &c. &c.

The *second,* cases of compound fractures of the extremities and amputations.

In this ward, there was one curious, but, alas! by no means uncommon case, of spontaneous amputation of both legs. The wretched object was one of the sufferers from fever at Vittoria, and one of the consequences of that fever, and of the cold and wet to which he and his companions were inhumanly, and, in a great degree, unnecessarily exposed, by the Spanish jealousy of Cordova, was, mortification of both feet, a little above the ankles—the feet literally dropping off, and the vis medicatrix naturæ supplying their place with very fair stumps, without the aid of surgeon or surgical instrument.

It would be unjust to those who have had the anxiety and labour of treating the cases in this ward, and from whom it is expected that some valuable practical observations are forthcoming, were the writer to cite particular cases of interest here. The number of compound fractures of the extremities has been very great, and the treatment generally very successful. In cases of fracture within the capsular ligament of the femur, several attempts, however, have been made to save the limb, but always, I believe, at the cost, ultimately, of the life. The possibility of re-union of the broken bone within the capsule, is still however insisted upon, and apparently somewhat countenanced by the result in one femur which was shewn me, and the history of which, it is thought, will be published by Mr. Alcock—but, in that example, the re-united portion of bone had not been fractured within, but at the insertion of, the ligament. And the practice is surely perilous in the extreme to the patient, protracting, of necessity, his sufferings to so great a length, as to leave him, at last, little chance of deriving benefit even from an operation. While the advantage to be gained in the preservation of a limb, however desirable, cannot for a moment be weighed in the balance against the fearful loss of life, which has hitherto attended such attempts to restore the soldier to the service without mutilation. The knife is the dernier resort certainly of the surgeon whose head and heart are alike accomplished for the successful exercise of his profession, one with knowledge and judgment, and the other with sensibility and kindness. It is as certainly the first appeal of the inexperienced and unfeeling, because ignorant, novice in surgery. But, when resorted to, as in the cases referred to, we contend it ought to be, it should be with promptitude and without fear.

I may here also take occasion to observe, that the various divisional surgeons at San Telmo, have, in their amputations, adopted very different practices. One continues the old circular, and, we may say, circuitous method—another, treading in the steps of his master, Mr. Liston, has introduced the flap operation. A third has attempted to improve it by an operation, half-circular half-flap, preferable to the common circular one in the subsequent appearance of the stump, inferior to that of the flap by prolonging the operation, and consequently the punishment of the patient.

The *third* division or ward is given up to cases of complicated flesh-wounds;—wounds penetrating the joints and great cavities, or accompanied with lesions of arteries and nerves;—and, compound fractures of the head and chest. The cases here, as will readily be conceived, were of great interest, and some of them exceedingly curious. The great advantage of constant medical superintendance, was apparent in the recovery of some, at first hopeless, cases of gunshot wounds penetrating the lungs and fracturing the ribs. Indeed it is impossible that any thing can exceed the attentions paid by the divisional surgeons to their respective wards—and to the comfort and well-being of the men entrusted to their care. The happy results will be manifest should the proportional tables which are kept, be hereafter published.

The FOURTH and FIFTH divisions are reserved wards, used chiefly for convalescents.

And, a further subdivision of cases is obtained by arranging corresponding cases in sets.

Each division has one staff surgeon, and two assistants. The average number of patients in each ward, for whom professional attendance is thus provided, is about 90, or 450 in the whole hospital, which, however, has, upon occasions, contained upwards of 500 cases at one time.

Under the medical officers of each division, the following servants are employed; 1 female nurse, 1 ward master, an assistant ward-master, who acts as clerk of the division, and ten orderlies, these last, being more or less, disabled men.

The orderlies are selected principally from " the hospital corps," which was formed of men who had been previously declared unfit for regimental duty by a board, composed of three staff-surgeons, with, now and then, the inspector, or Deputy-Inspector-General, and consists of 100 men for service in the interior of the hospitals, at an average of one orderly to eight patients. They are paid by the Deputy Purveyor 1*s.* 1*d.* per diem, and commanded by a Lieutenant.

Besides this corps, there is another, called the " Hospital Transport Corps," commanded like the former, by Lieut. Smith, B.A.L.; but, unlike the former, composed of able-bodied men, from 30 to 40 originally, since increased to 50, and mostly tradesmen. This corps was formed in England—is employed on fatigue duty, and in repairing defects about the building, &c. They are paid like the wagon-train, 1*s.* 10*d.* per diem, and supply the wardsmen.

The Staff-Assistant Surgeons perform the duties of " Orderly Medical Officer" in rotation, when they remain throughout the day, and also sleep in the hospital in readiness to receive casualties, and prescribe for cases of emergency. An admirable plan, which might, with some modification, be adopted in our metropolitan hospitals—and is, or, at least was, acted upon, at the London Hospital. The advantage to the students in such case, being obvious, interesting them in particular cases—and accustoming them to act subject to the correction of others, before sending them forth to act altogether upon their own responsibility.

Attached to each Division is an Orderly Room, where the various reports are made up—and in which the respective duties of every servant in the establishment are accurately defined, and explicitly stated.

The beds were ranged in three parallels along each ward, somewhat crowded, of necessity—but all, as cleanly as could be expected.

On the arrival of every new patient, his clothes, &c. are exchanged for a complete suit of hospital clothing, and, together with his arms and accoutrements, deposited in a store, which, for neatness and order may vie with any thing of the kind in England. It is a part of the ward-master's duty before taking down a patient's clothing, to stitch the name of the owner to each article, with their joint initials attached, an excellent and necessary precaution against suspicion of robbery or fraudulent exchange, when restored.

The patients are discharged twice a week, at which times the surgeons of divisions present to the deputy inspector-general a list of such as they deem meet to be sent out, and he further examines such men, and either confirms the previous opinion, or remands them for ulterior treatment.

The foregoing notice has been confined to the hospitals belonging to the British Auxiliary Legion, and is almost a literal transcript of notes made on the spot at different visits paid by the writer, on his occasional arrivals at San Sebastian in one of his Majesty's vessels of war. That it is so meagre he regrets exceedingly—but the condition of a naval medical officer, particularly if alone, is of all conditions the most precarious, he can neither calculate upon time or tide, wind or weather—and such facts as he can collect, he must gather as he can, not as he would, happy, if allowed to gather any beyond the precincts of a midshipman's berth if an assistant surgeon—or, of a gun-room, if a surgeon.

And this remark suggests to the writer a mode of accounting for, what, to himself, appeared strange, and to others was *felt* as ungracious, not to say ungenerous, in his professional brethren employed on the North Coast of Spain. Only one or two had ever visited the Hospital of San Telmo. Even the second surgeon of marines, living as he did at San Sebastian, had never "honoured" the interior of that building with his presence—and thus let slip an opportunity for which others would have gladly paid any price. Why this apparent apathy—why this "tipping the cold shoulder" to members of the same profession—arduously, and, truly, honourably engaged, in the peaceful and laborious exercise of that profession? It may seem abundantly absurd, but it is nevertheless unquestionably true that the distinction of caste holds elsewhere than in India. The Officers of his Britannic Majesty's Navy deem it derogatory in them to associate generally with the Officers of her Catholic Majesty's British Auxiliary Legion—and it is feared that the medical corps of that navy participate in such narrowness of feeling. But this is not the case. The Hospital at San Telmo would, doubtless, have been more generally, and frequently visited by the medical men of the squadron—had their stay not been so uncertain as it invariably is, in harbour—and were the difficulties to civilians going to and fro out of their ships less than they are rendered by those in command.

It may now be added that, there being several hundred royal marines employed at Passages, a port distant three miles from San Sebastian—and a good number of royal marines, royal artillerymen, and engineers, engaged strengthening the lines between those places—there are also two surgeons R.N. attached to them—who reside on shore, the senior, Mr. Dabbs, at Passages, where he has a very well regulated hospital. His whole time is nearly occupied in personal attendance upon the arduous and excessive duties of an hospital, for the sick and hurt of upwards of 800 men—without even an assistant being allowed him, while his colleague at San Sebastian, has, comparatively, nothing to employ him, and, being of equal rank, cannot, and ought not, consistently with official etiquette, to be employed as an assistant. Here, at Passages, the diseases have been chiefly visceral—and epidemic—partly the effect of climate—partly of the local situation of the town—and partly also of the incessant occupation of the men on the summit of a hill, which bares its head to every Northern blast—and on which, for many months, there was nothing worthy the name of shelter either from wind or rain.

The Spanish Hospitals have not been noticed—but little sympathy appears to exist between the Spanish and English faculties—and little correspondence, as a matter of course, takes place between them. What was, in peaceable times, the Esculas Publicas, or Public School, is now an hospital for Spanish sick and hurt.—I visited it, but could find no medical guide through the wards, which were clean and quiet—and was obliged to content myself with the exchange of civilities, and with pouring a word of comfort and encouragement here and there upon the auditory nerves of our Spanish neighbours."

